# The Interplay Between Juvenile Delinquency and ADHD: A Systematic Review of Social, Psychological, and Educational Aspects

**DOI:** 10.3390/bs15081044

**Published:** 2025-08-01

**Authors:** Márta Miklósi, Karolina Eszter Kovács

**Affiliations:** 1 Institute of Education and Cultural Sciences, Faculty of Humanities, University of Debrecen, Egyetem tér 1, 4032 Debrecen, Hungary; miklosimarta@unideb.hu; 2 Department of Counselling, Developmental and School Psychology, Institute of Psychology, Faculty of Humanities, University of Debrecen, Egyetem tér 1, 4032 Debrecen, Hungary

**Keywords:** ADHD, juvenile delinquency, comorbidity, risk factors, early intervention

## Abstract

Attention deficit/hyperactivity disorder (ADHD) is a neurodevelopmental disorder characterised by inattention, hyperactivity, and impulsivity, frequently observed in juvenile offenders. This systematic review explores the interplay between ADHD and juvenile delinquency, focusing on behavioural, psychological, and social dimensions. Following the PRISMA guidelines, a systematic literature review was conducted using EBSCO Discovery Service, Science Direct, PubMed, and snowballing techniques. Studies meeting specific inclusion criteria, including juvenile offenders diagnosed with ADHD and comparisons to non-offender or non-ADHD control groups, were analysed. The methodological quality of studies was assessed using the Joanna Briggs Institute appraisal tools. A total of 21 studies were included, highlighting significant associations between ADHD and juvenile delinquency. ADHD symptoms, especially impulsivity and emotional dysregulation, were linked to an earlier onset of offending and higher rates of property crimes. Comorbidities such as conduct disorder, substance use disorder, and depression exacerbated these behaviours. Sociodemographic factors like low education levels and adverse family environments were also critical modifiers. Early intervention and tailored treatment approaches were emphasised to address these challenges. The findings underscore the need for early diagnosis, individualised treatment, and integrative rehabilitation programmes within the juvenile justice system to mitigate long-term risks and promote social inclusion.

## 1. Introduction

Attention deficit/hyperactivity disorder (ADHD) is a mental condition linked to nervous system dysfunction, primarily characterised by hyperactivity, impulsivity, and attention deficit (Sarver et al., 2014). ADHD can be attributed to both genetic and environmental influences. Studies examining hereditary factors indicate that first-degree relatives face a 3–5-times-higher risk of developing ADHD, while twins show a risk ranging from 65 to 90% (Faraone et al., 2000). In the early 1900s, the initial understanding of ADHD suggested it stemmed from disruptions in moral controls and related motivational issues that were often apparent to those around them (Mattiassich-Szokoli & Sófi, 2022). Thanks to Faraone’s research, it has become increasingly clear that ADHD has a clear molecular biological and genetic background in addition to environmental risks (Faraone et al., 2000). A study conducted by Coolidge et al. (2000) demonstrated that ADHD has an average heritability rate of 76%, ranking it among the most genetically inherited psychiatric disorders. Additionally, factors such as complications during birth, maternal smoking, and familial issues may also play a role (Eaves et al., 2000; Savolainen et al., 2010). Adverse childhood experiences (ACEs) may also be relevant factors, as ACEs are common among juvenile offenders. Laajasalo et al. (2025) found that youth involved in the juvenile justice system were more than 12 times more likely to have experienced at least one ACE than their uninvolved peers. Research confirmed that they may influence the development of ADHD and the onset and maintenance of delinquency (De Sanctis et al., 2012).

Childhood ADHD prevalence estimates range between 4 and 12% (Cornish et al., 2005), and follow-up research indicates that 70–80% of those diagnosed continue to exhibit symptoms into adolescence (Eaves et al., 2000; Savolainen et al., 2010). The prevalence rate is two to four times greater in males than in females (Barkley, 2002; Barzman et al., 2004; Biederman & Faraone, 2005). These differences in gender are noticeable at an early age but tend to diminish as individuals grow older. Clinical studies reveal that boys display a higher incidence of hyperactivity–impulsivity than girls (Barkley, 2002; Barzman et al., 2004). Additionally, meta-analyses show ADHD prevalence rates of 26–30% among juvenile and adult detention populations, reflecting a risk that is five to ten times greater than that of the general population (Baggio et al., 2018).

Disruptive behaviour patterns are often exhibited by children with elevated levels of ADHD, especially during their teenage years, indicating that hyperactivity might serve as a predictor for antisocial behaviour (Babinski et al., 1999). While numerous studies have shown that children diagnosed with ADHD face a heightened risk of delinquency, the clarity surrounding ADHD’s role in predicting delinquency in both clinical and population-based studies remains inadequate (Barkley, 2002; Mannuzza et al., 2008). Barkley’s research revealed that children with ADHD were more inclined to partake in antisocial activities than their peers and emphasised that the frequency of these atypical behaviours was a strong indicator of ADHD severity during childhood and adolescence (Barkley et al., 2004).

A study in Finland found that more than 50% of prison inmates met the diagnostic criteria for ADHD, compared to 45% in Germany (Haapasalo & Hämäläinen, 1996; Rösler et al., 2004). In a Korean study evaluating 98 juvenile detainees and 84 non-offending controls, 42.4% of adolescents with a history of delinquent offending were diagnosed with ADHD, compared to only 11.9% of the control group (Chae et al., 2001).

While the research results are clear, the mechanisms that might connect ADHD with delinquency remain largely unexplored (Thapar et al., 2006). Caution is warranted when considering these connections, as hyperactivity and attention deficit frequently co-occur with other early risk factors, including conduct disorder, familial disadvantage, and low verbal intelligence (Savolainen et al., 2010). Research has established the link between ADHD and juvenile delinquency, with studies showing that children and adolescents diagnosed with ADHD are at higher risk of delinquency than those without ADHD, particularly in the presence of comorbid conduct disorder (Foley et al., 1996; Sibley et al., 2011). The core symptoms of ADHD (impulsivity, inattention, and hyperactivity) can contribute to poor decision making and difficulty complying with rules, which increases the propensity to delinquent acts (Foley et al., 1996; Forehand et al., 1991; Sibley et al., 2011). Hence, the mechanisms of the link between ADHD and delinquency are likely to include a lack of reasoned decision making, impulsivity, inattention, higher risk taking and hyperactivity, and a higher risk factor for committing violent acts. These conditions contribute to poor decision making, rule-breaking, emotional dysregulation, and executive dysfunction (Royal College of Psychiatrists, 2023). Different ADHD symptom profiles can lead to different patterns of offending; for example, impulsive ADHD is associated with impulsive crimes such as robbery, while inattentive ADHD is associated with more premeditated crimes (Royal College of Psychiatrists, 2023). Additionally, the influence of ADHD on delinquency may be shaped by associated negative factors, such as educational failure, which is commonly observed in the lives of young individuals affected by this condition (Rösler et al., 2004; Thapar et al., 2006).

Research by Moffitt emphasises that the transition from youth to adulthood represents a crucial phase concerning the age crime curve. He differentiates between life-course-persistent offenders and adolescence-limited offenders in his analysis of severity and continuity, noting that juvenile delinquency is typically the norm rather than an anomaly (Moffitt, 2003). According to Moffitt’s dual taxonomy, the distinction between life-course-persistent offenders and adolescent-restricted offenders lies in the early emergence of antisocial behaviour (Moffitt & Caspi, 2001). The main contributors to adult delinquency are neuropsychological deficits, which may be inherited or developed during early childhood. It is suggested that growing up in a challenging family setting heightens the criminogenic potential of these risk factors (Moffitt, 2003). Consequently, ADHD is viewed as a reflection of a neuropsychological deficit that could lead to a chronic and persistent trajectory toward delinquency (Farrington, 2003). Moffitt (1990) proposed that early risk factors directly influence delinquency in adulthood rather than being influenced by social factors during later adolescence. While the theory acknowledges that hyperactivity and impulsivity can lead to adverse outcomes throughout life, it views these results as downstream effects—distinct expressions of a shared syndrome—that contribute minimally to the overall causal sequence (Savolainen et al., 2010).

Sampson and Laub (1993) proposed that the majority of antisocial children do not transition into criminality, asserting that an antisocial upbringing is not a prerequisite for delinquency in adulthood. Adhering to the fundamental principle of social control theory, they contend that weak connections to prosocial figures lead to delinquent behaviour (Sampson & Laub, 1993). Consequently, Farrington (2003) posits that while ADHD is not a definitive cause of adult delinquency, it serves as a risk factor that may influence delinquent behaviour by obstructing robust ties to conventional institutions.

During the teenage years, the primary sources of social control are attachments to parents, peers, and school, and issues related to hyperactivity and inattention can significantly impact these connections. Furthermore, research indicates that children diagnosed with ADHD are less frequently raised by both biological parents (Foley et al., 1996) and that their parents often face challenges such as low socioeconomic status and criminal histories (Farrington, 2016). Such traits are typically linked to diminished levels of social control (Eaves et al., 2000). Additionally, irrespective of the characteristics of the parents, the tendency of ADHD to manifest as poor interpersonal skills likely affects parenting styles and socialisation practices (Setyanisa et al., 2022). Research has shown that inadequate parental discipline, low-quality parent–child relationships, and weakened family cohesion exacerbate the connection between ADHD and antisocial behaviours (Thapar et al., 2006).

Based on the literature review, we can state that juvenile offenders are a very complex population, and it becomes important to have a global vision of the adolescent, including family and individual risk factors. This systematic review aims to shed light on the interplay between juvenile delinquency and ADHD through exploring behavioural, social, and psychological aspects. The review considers some traits associated with the disorder, such as emotional dysregulation and impulsivity, but neglects the early onset of criminal behaviour as a fundamental aspect. Furthermore, we make an attempt to analyse the additional factors such as conduct disorder, substance abuse, and depression that accompany the disorder and increase the propensity to delinquency. Several family or sociodemographic variables, including education, employment status, and parental support, are also believed to be of great importance in modifying the relationship between ADHD and delinquency. Also, the treatment needs of the patients are highly dependent on the mechanisms hypothesised to mediate the relationship between ADHD and criminality, thus calling for individualised and holistic treatment approaches.

## 2. Materials and Methods

**Figure 1:**
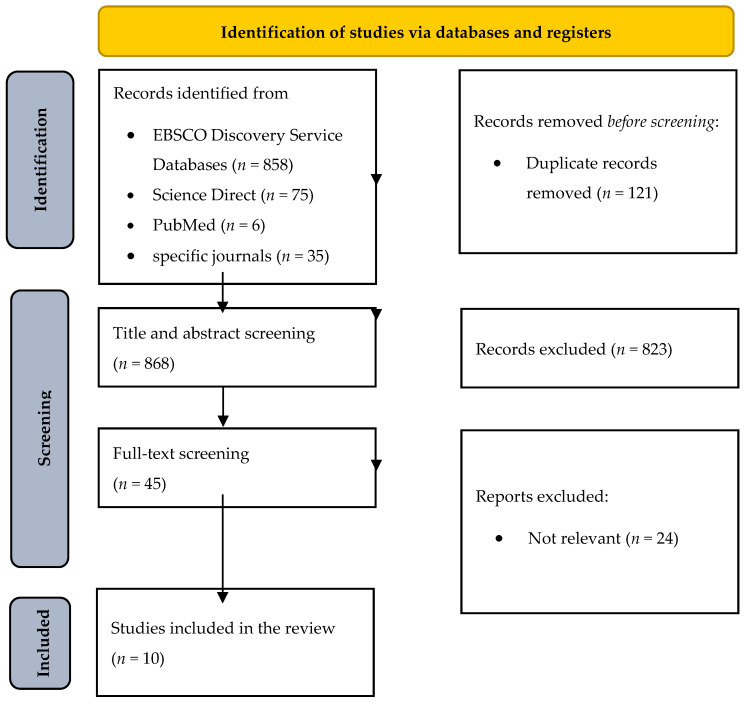
Preferred Reporting Items for Systematic Reviews and Meta-Analyses (PRISMA) diagram.

This systematic literature review was created based on the Preferred Reporting Items for Systematic Reviews and Meta-Analyses (PRISMA) guidelines (Moher et al., 2015, see Figure 1).

### 2.1. Literature Review

The EBSCO Discovery Service Search Engine, Science Direct, and PubMed were used for systematic search. Using the Boolean search string, the following keywords we applied for searching were as follows:

(“juvenile delinquency” OR “juvenile offenders” OR “youth offenders” OR “juvenile justice” OR “juvenile corrections”)

AND

(“ADHD” OR “attention deficit hyperactivity disorder” OR “attention deficit-hyperactivity disorder”).

Beside the search engine, snowball search was applied, checking the reference list of papers found by the search engine. Also, the top 30 journals indexed in Web of Science (having the highest percentile above 50%) were screened (see Appendix A, Table A1). Journals were selected based on their indexing in Scopus and Web of Science and their impact metrics, including citation percentile and relevance to the fields of psychology, psychiatry, education, and criminology. The searches were performed in August 2024. Unscreened articles were listed in Zotero (V6.0.22, Roy Rosenzweig Center for History and New Media, George Mason University, Washington DC, USA).

### 2.2. Inclusion and Exclusion Criteria

The following inclusion criteria were set, following the PICOS format (P: population; I: intervention; C: comparison; O: outcome; S: study design):

- *Population*: Juvenile offenders/criminals;
- *Intervention*: Original empirical research published in a peer-reviewed journal;
- *Comparison*: Examined juvenile offenders diagnosed with ADHD compared to those without ADHD diagnosis or with comorbid issues in various contexts (sociodemographic background, nation, psychological characteristics, or non-offenders as a control group);
- *Outcome*: Behavioural outcomes and criminal offending and academic achievement;
- *Study design*: Observational, interview, survey, cohort study, or randomised controlled trial.

Papers had to be written in English, published between 2004 and 2024, and in the disciplines of psychology, social sciences, humanities, and educational sciences. Language restrictions have been applied to ensure the consistent application of quality appraisal tools and to minimise the risk of misinterpretation due to translation inaccuracies. Review papers, commentaries, letters to the editor, conference papers, books, book chapters, dissertations, or newspaper articles were excluded. Grey literature (e.g., dissertations, conference proceedings, and government reports) have also been excluded to maintain a focus on peer-reviewed, methodologically rigorous studies.

### 2.3. Data Extraction and Assessment of Methodological Quality

A comprehensive multistage screening procedure was implemented to identify studies meeting the inclusion criteria. The authors independently searched the literature and examined the titles and abstracts of every study. Following this, all identified records were subjected to a screening of their titles and abstracts. Studies that met the inclusion criteria then proceeded to an exhaustive full-text evaluation. The authors oversaw the meticulous analysis, quality assessment, and data extraction of the chosen studies. In cases of ambiguity, discussions among the authors were held to arrive at a consensus.

An Excel spreadsheet along with data extraction forms were used for data extraction. We included the full article citation, sample characteristics (number of participants, gender, ethnicity, diagnosis of ADHD, comorbid disorders), aim of the paper, methods (qualitative or quantitative), tools applied, results/outcome, and comments related to study quality. Most studies carried out cross-sectional research regarding the methodological framework. Some studies conducted long-term follow-ups, up to 15 years, to examine recidivism and crime patterns. Some studies combined data from interviews, questionnaires, and official criminal records. Therefore, the risk of bias and quality of the studies was evaluated using the Joanna Briggs Institute (JBI) critical appraisal tool, focusing on cross-sectional studies (Moola et al., 2015), qualitative studies (Lockwood et al., 2015), case series (Munn et al., 2020), and randomised controlled trials (Barker et al., 2024). Each article was assessed with the appropriate tool on a 4-point scale (yes/no/unclear/not applicable).

## 3. Results

Overall, 989 records were detected. After double filtering, 121 records were excluded, and after title and abstract screening, 823 records were excluded. During the title and abstract screening phase, the majority of the 823 excluded records were omitted because they (a) did not focus on juvenile offenders, (b) did not address ADHD explicitly, (c) were review articles or grey literature, or (d) lacked empirical data relevant to our outcomes of interest. Therefore, 45 papers were sent for full-text screening, which led to the involvement of 21 papers in the qualitative synthesis.

The studies have been conducted in various countries, including Germany (Barra et al., 2022; Grieger & Hosser, 2012; Kaplan & Cornell, 2004; Philipp-Wiegmann et al., 2018; Retz et al., 2004), the United States of America (Khanna et al., 2014; Sibley et al., 2011; Silva et al., 2014; Wojciechowski, 2021), Hong Kong (Poon & Suk-Han Ho, 2015), Russia (Lindblad et al., 2020), South Korea (Cho et al., 2013), the Netherlands (Rutten et al., 2022), India (Garg et al., 2024), Sweden (Ståhlberg et al., 2017), and Nigeria (Atilola et al., 2021).

### 3.1. Sociodemographic Factors

The study’s measurement of the relevance of sociodemographic factors was under-represented. Regarding gender, most studies measured only male offenders (Atilola et al., 2021; Garg et al., 2024; Grieger & Hosser, 2012; Kaplan & Cornell, 2004; Khanna et al., 2014; Lindblad et al., 2020; Philipp-Wiegmann et al., 2018; Poon & Suk-Han Ho, 2015; Retz et al., 2004; Rutten et al., 2022; Sibley et al., 2011). Studies investigating only female offenders did not appear. Focusing on both sexes without any comparisons was typical in two cases (Sarver et al., 2014; Ståhlberg et al., 2017), while the investigation of gender differences was unclear in three studies (Cho et al., 2013; Jones et al., 2021; Margari et al., 2015). Gender comparison appeared in two studies (Barra et al., 2022; Silva et al., 2014; Wojciechowski, 2021). In the study of Barra et al. (2022), male participants were over-represented in the low ADHD subtype, while females were over-represented in the severe ADHD subtype. They also reported that young women who committed offences tended to exhibit more severe ADHD, with or without intermittent explosive disorder (IED), along with additional behavioural issues; however, there were no significant differences noted in terms of cumulative adverse childhood experience (ACE) burden. Silva et al. (2014) reported no significant difference in the average age of initial incarceration for both boys and girls. When compared to the control group, those with ADHD, regardless of gender, were twice as likely to engage in such offences. While boys and girls exhibited a comparable pattern of first offences, the data for girls were limited. Children diagnosed with ADHD, both boys and girls, are notably more prone to having records of community corrections and incarceration compared to those without the disorder. While boys with ADHD were more inclined to have a community correction record at an earlier age, this trend was not observed in girls. However, the highest number of offences occurred among individuals aged 15 to 17.

In addition, only a few studies focused on social status and family-related factors. The study of Wojciechowski (2021) exclusively focused on the severity of violent behaviour in light of sociodemographic factors and ADHD. The results stated that individuals who qualified for an ADHD diagnosis at the baseline were most likely to be placed in the High Chronic violent offending category. The probability of being categorised into the Desisting and Moderate Stable trajectory groups, as opposed to the Abstaining group, was notably increased for individuals who met the ADHD diagnostic criteria at baseline. Additionally, having an ADHD diagnosis at baseline not only predicted involvement in violent offences but also indicated a potential increase in the frequency of offences committed by juvenile offenders. When it comes to gender, males exhibited a significantly higher likelihood of being assigned to the Moderate Stable, Desisting, and High Chronic groups compared to the Abstaining group. In relation to race, only the assignment to the Moderate Stable trajectory was significantly affected, with Black participants facing a considerably greater relative risk of being placed in this trajectory group than their White counterparts. Socioeconomic status (SES) did not significantly influence the relative risk of assignment to any of the trajectory groups.

Rutten et al. (2022) revealed disparities among adolescents concerning comorbidity, education, and living conditions. Comparing juvenile offenders diagnosed with ADHD and/or ASD, the group diagnosed solely with ADHD demonstrated a lower level of completed education and had fewer adolescents living with both biological parents. Retz et al. (2004) stated that individuals with ADHD were notably younger, had lower educational attainment, and experienced higher unemployment rates when compared to those without ADHD. Additionally, the age at which they first faced conviction was younger, and the incidence of delinquent behaviour before reaching the age of criminal discretion was greater than that observed in young prisoners who did not have ADHD.

Ståhlberg et al. (2017) investigated how psychosocial background elements, such as age at first conviction and substance abuse or dependence on primary relatives, along with clinical factors like ADHD diagnosis, IQ score, and the age at which drug abuse began, influence the persistence of violent criminal behaviour. Their findings indicated that age at first conviction plays a significant role in persistent violent criminality, suggesting that an earlier first conviction correlates with an increased risk of developing ongoing violent behaviour.

The study of Barra et al. (2022) highlighted that many young offenders carry the weight of adverse childhood experiences. Over 85% of those surveyed reported encountering at least one of the five evaluated ACEs, while more than 28% stated they had faced at least four out of the five categories. High ADHD severity was related to increased ACE rates; the cumulative ACE score predicted severe ADHD, but only when coexistent with IED. Therefore, elevated levels of ACEs are found not only among intensive and chronic young offenders but also among those delinquents who are in the early phases of their criminal development.

### 3.2. Type of Crime

Offences can be grouped according to several criteria, one of which is the distinction according to the object of the offence. Richards (2011) points out that the police more often arrest juveniles for crimes against property than for crimes against a person. In particular, according to researchers, “property crime, carrying a concealed weapon, illegal drug possession, and arrest rates have been shown to be positively related to ADHD status” (Biederman & Faraone, 2005; Fletcher & Wolfe, 2009; Mannuzza et al., 2008).

The predominance of crimes against property has been highlighted in some research (Barra et al., 2022; Grieger & Hosser, 2012; Lindblad et al., 2020; Philipp-Wiegmann et al., 2018; Rutten et al., 2022; Silva et al., 2014). Philipp-Wiegmann et al. (2018) found that boys with ADHD were more likely to be involved in non-violent property crimes (property crime = 58.6%) than crimes against the person (assault = 21.8%). Similar results were found by Barra et al. (2022) (crimes against property = 35.9%; assault = 21.8%) and Grieger and Hosser (2012), although the difference was smaller (crime against property = 34.8%; assault = 33.3%). Rutten et al. (2022) found a significant correlation in their study, showing that the proportion of non-violent crimes against property was significantly higher among people with ADHD (22, 24%) than among people with ASD. When disaggregating the crimes, it was found that the most common crimes among the group of adolescents with ADHD were violent property crimes (37.8%), non-violent property crimes (24.4%), and moderate violent crimes (21.1%). Lindblad et al. (2020) also found in their study that most juveniles were convicted of crimes against property (theft, car theft, etc.; 51%), followed by crimes related to violence (e.g., assault, robbery; 38%), and the smallest proportion of juveniles in the sample were convicted of sexual violence (6%) or homicide (5%). In their study, Silva et al. (2014) categorised offences into eight groups, but of these, burglary was also the most common, with this offence being twice as likely in boys with ADHD as in boys without ADHD.

A study conducted by Margari et al. (2015) revealed contrasting findings, indicating that juvenile offenders responsible for crimes against individuals exhibited a higher prevalence of ADHD symptoms (18%) and behavioural issues (20%) compared to those involved in property crimes as well as alcohol- and drug-related offences. Similar results were found in research by Kaplan and Cornell (2004), comparing instrumental violent crime (violence used as a means to achieve an external goal, e.g., robbery) and reactive violent crime (motivated by anger, revenge, or frustration, in which the goal of the violent act is primarily to harm the victim). His research found that the juveniles studied were more likely to have committed reactive violent crime (*n* = 50) than instrumental violent crime (*n* = 39).

In their research, Atilola et al. (2021) highlighted a particularly intriguing outcome regarding the types of crime in Africa, revealing that over two-thirds of the juveniles analysed were apprehended for status offences—actions deemed non-criminal that are classified as violations solely due to the offender’s minor status. These offences encompassed truancy, running away from home, vagrancy, violations of curfew, associating with dangerous adults, and consistently disregarding household rules. This finding aligns with comparable studies in Nigeria (Atilola et al., 2017; Issa et al., 2009; Olashore & Olashore, 2017) across Africa (Munene & James, 2017).

### 3.3. Age of Onset

Of the studies reviewed, five examined the relationship between age at first offence and involvement with ADHD. The studies found similar results, suggesting that there is consistent evidence that individuals with ADHD start offending at a younger age.

Retz et al. (2004) found that ADHD offenders have a lower average age at first arrest and a higher rate of offending at age 14 compared to juveniles who do not have ADHD. In their study, Ståhlberg et al. (2017) conducted an ROC (receiver operating characteristics) analysis to evaluate the continuous variable of age at first conviction in relation to the persistence of violent criminality, aiming to enhance its predictive accuracy. This relatively straightforward variable significantly predicted the continuation of violent criminal behaviour, achieving an AUC of 0.69 (AUC = area under the ROC curves).

A study conducted by Sibley et al. (2011) revealed that boys diagnosed with childhood ADHD alone and those with both childhood ADHD and ODD faced a heightened risk of delinquency by age 18. These two groups were notably more inclined to have committed prior offences, recorded more offences, and engaged in more severe offences than their juvenile counterparts. The implications of these findings indicate that boys with ADHD who displayed minimal antisocial behaviour during primary school still face the potential for future criminal activity. This concern is significant, as early delinquency and engagement in various crimes correlate with an atypical delinquency trajectory (Mazerolle et al., 2000; Moffitt, 2003). Additionally, this outcome aligns with other research indicating that adolescents with ADHD tend to begin committing crimes at an earlier age than those without the condition (Forehand et al., 1991; Moffitt, 2003). Regarding serious delinquency, both boys with ADHD only (23.4%, OR = 1.84) and those with ADHD + ODD (25.4%, OR = 2.01) exhibited comparable levels of risk.

According to Silva et al. (2014), males diagnosed with ADHD engaged with the justice system for the first time at an earlier age than those in the control group. Typically, the average age for their initial involvement with community correction or incarceration ranged from 15 to 17 years. Among the children with ADHD who appeared in the community correction register, 308 boys (39%) were aged between 10 and 14 years, whereas the control group had 235 boys (29%) in the same age range. Boys with ADHD received their first community correction record at a younger average age compared to their counterparts in the control group, with ages of 15 years and 9 months versus 16 years and 3 months.

DeLisi et al. (2013) examined differences in the onset of ADHD between young people with and without the disorder. Youths with ADHD showed significantly earlier antisocial onset than youths without ADHD. Children with ADHD broke the rules for the first time 1.1 years earlier, had their first contact with the police 1.3 years earlier, and had their first juvenile court appearance 0.8 years earlier.

### 3.4. Psychological Consequences

Some studies have used a diagnostic criteria system (DSM-IV, DSM-5, ICD-10) to identify ADHD, ODD, CD, or other comorbid disorders (Atilola et al., 2021; Chung et al., 2011; Margari et al., 2015; Rutten et al., 2022; Sarver et al., 2014; Sibley et al., 2011; Silva et al., 2014; Ståhlberg et al., 2017). In addition, diagnostic interviews (e.g., K-SADS) have been used in some cases (Khanna et al., 2014; Lindblad et al., 2020). However, several studies have used self-completion questionnaires to map behavioural and psychological characteristics, even for ADHD categorisation (Barra et al., 2022; Cho et al., 2013; Garg et al., 2024; Grieger & Hosser, 2012; Philipp-Wiegmann et al., 2018; Poon & Suk-Han Ho, 2015; Retz et al., 2004). In the case of some studies, the diagnostic criteria for assigning offenders to the ADHD group were unclear, making it difficult to determine how participants were classified (Atilola et al., 2021; Kaplan & Cornell, 2004; Wojciechowski, 2021).

Barra et al. (2022) reported that symptoms of ADHD were notably common, with one-fourth of the participants indicating at least moderate symptoms and another quarter experiencing severe symptomatology. The ratio of participants with ADHD was high in the research of Cho et al. (2013) too; subjects with ADHD constituted 32.4% of the total participants.

Barra et al. mentioned that increased rates of additional internalising and externalising issues were associated with the high severity of ADHD, emphasising the range of psychiatric disorders that often accompany it. Therefore, when considering further psychiatric conditions, it is crucial to take into account not just ADHD but also the presence of coexisting IED symptoms. The results of Retz et al. (2004) also support these findings. In their research, offenders belonging to the ADHD group exhibited significantly higher scores for internalising issues, such as anxiety, depression, social withdrawal, and somatic complaints. Additionally, the personality dimension of “neuroticism” (measured by the Five-Factor Inventory) showed higher scores, indicating that these subjects were more anxious, depressed, and vulnerable while also displaying increased levels of hostility, self-consciousness, and impulsivity compared to the control group, reinforcing the same conclusion. This was also supported by the results of Garg et al. (2024), who found a significant difference between juvenile offenders with and without ADHD concerning the severity of their conduct issues. The level of conduct issues was also influenced by the existence of oppositional defiant disorder (ODD). Margari et al. (2015) also focused on the evaluations of emotional and behavioural issues, which indicated that juvenile offenders experienced both externalising (31%) and internalising (23%) challenges. Internalising issues were primarily characterised by withdrawn or depressed symptoms (13%), followed by anxiety/depression (12%), somatic problems (10%), and affective issues (9%). On the other hand, externalising problems showed huge variation concerning the diagnosis, and they were predominantly represented by oppositional defiant behaviours (19%), followed by rule-breaking actions (13%), conduct issues (11%), and ADHD (10%). These findings align with previous studies that noted a greater prevalence of externalising behaviours, such as conduct disorder, ADHD, and substance abuse, among adolescent offenders compared to typical internalising mental health concerns like depression, panic disorder, and anxiety. This also had a significant impact on close relationships. Children with ADHD experienced a higher rate of peer rejection. They reported fewer close friendships in comparison to those without ADHD, indicating that the long-term impact of ADHD on social interactions is particularly significant for youngsters who continue to have ADHD or exhibit conduct disorder during their teenage years.

The results of the studies focusing on recidivism were ambivalent. Grieger and Hosser (2012) found that the occurrence of ADHD among prisoners was significantly greater than the prevalence estimates obtained from community samples. Over 50% of the inmates fulfilled the criteria for having ADHD during their childhood. However, in summary, their study’s findings do not confirm the hypothesis that ADHD can forecast criminal recidivism. The analyses conducted did not indicate that ADHD had any effect on survival curves or the likelihood of reoffending. However, the results of Philipp-Wiegmann et al. (2018) discovered the significant role of ADHD as a risk factor for recidivism since, based on the results of the 15-year-long follow-up study, offenders diagnosed with ADHD had a significantly faster recidivism rate (2.5 times less time to the next reconviction compared to the offenders without ADHD). The number of further offences and re-incarcerations was also higher in the ADHD subgroup. This study also revealed that ADHD moderates both the relapse and the course of delinquency concerning the number of further engagements in the legal system.

### 3.5. Comorbid Disorders

Depression also appeared as a comorbid disorder. In the study of Cho et al. (2013), the group experiencing depression was significant, representing 52% of all subjects. Symptoms of ADHD demonstrate a significant positive correlation. A noteworthy negative relationship was found between self-esteem and depression, indicating that lower self-esteem is associated with higher levels of depression. Perceived health state, ADHD symptoms, and self-esteem were assessed as independent variables, while depression served as the dependent variable. Together, these variables explained 37% of the variance in depression, with self-esteem identified as the most significant predictor. The relevance of depression was also measured by Chung et al. (2011), who stated that conduct disorder was not influenced by depression; however, it indicated that delinquent behaviour or conduct disorder might indirectly impact depression through factors like psychosocial impairment. A relevant correlation was identified between juvenile delinquency and depression and between self-esteem and juvenile delinquency. ADHD was shown as a contributing factor to delinquency, alongside depression, suggesting that the risk factors associated with juvenile delinquency have evolved over time. Compared to the control group, juvenile offenders showed significantly more ADHD symptoms, depression, anxiety, and suicidal ideation.

Obsessive–compulsive disorders are very often detected as comorbid disorders along with ADHD. The findings of Sibley et al. (2011) indicate that all children diagnosed with ADHD face an elevated risk for delinquency, irrespective of comorbidity. When compared to juveniles without ADHD, those with ADHD only, and those with ADHD and ODD, those with ADHD and CD exhibited a greater risk for every index of delinquency, including severity, variety, and age of initiation. The prevalence of severe offending was higher among children with ADHD only and those diagnosed with ADHD and ODD, who also faced a greater risk of starting mild and moderate delinquency at an earlier age, committing a wider range of acts compared to the comparison group. The risks demonstrated by the ADHD-only and ADHD and ODD groups were quite alike, differing only in the variety of offences committed.

Conduct disorder is also a frequently diagnosed comorbid disorder. In the study of Khanna et al. (2014), the group with CD/ADHD received higher ratings on individual factors of the Structured Assessment of Violence Risk in Youth (SAVRY) due to the inclusion of items such as ‘risk-taking/impulsivity’ and ‘attention deficit/hyperactivity difficulties.’ Additionally, they were rated significantly higher on lifestyle factors of the Psychopathy Checklist (PCL: YV), which features ‘stimulation seeking’ and ‘impulsivity’ as components. Furthermore, the CD/ADHD group scored notably higher on the social factor of the SAVRY, which encompasses aspects like peer rejection, ineffective parental management, stress, inadequate coping skills, and a lack of personal support. Lindblad et al. (2020) had similar results, revealing that ADHD is frequently encountered and almost invariably seen alongside CD. This particular diagnostic pairing was linked to elevated levels of comorbidity, more complex substance abuse issues, disruptive behaviours, and a significant prevalence of PTSD. The significant occurrence of PTSD linked to ADHD/CD carries a crucial clinical implication by emphasising the heightened risk of trauma exposure within this population. The current research indicates that individuals with a dual diagnosis of ADHD and CD exhibit greater levels of disturbance, particularly in terms of psychiatric comorbidities and heightened aggressive and disruptive behaviours.

Autism spectrum disorder (ASD) also appeared as a comorbid disorder in the studies analysed. Rutten et al. (2022) investigated juvenile offenders diagnosed primarily with ADHD and ASD, among whom 36.7% had ASD, 47.9% ADHD and 15.4% both ASD and ADHD. Besides these two disorders, disruptive behaviour disorder (36.2%), substance disorder (20.2%), and reactive attachment disorder (5.3%) appeared as comorbid disorders. In their study, no significant difference was found in the distribution of violent offences across diagnostic groups; however, individuals diagnosed solely with ASD exhibited a significantly higher likelihood of sex offending compared to those with ADHD, whether diagnosed alone or alongside ASD. In contrast, adolescents with ADHD faced a greater propensity for non-violent property offences than their ASD counterparts, whether diagnosed alone or in combination.

Intermittent explosive disorder was also investigated in one study. In the research of Barra et al. (2022), a significant occurrence of DSM-5-oriented IED (36%) was observed when compared to rates documented in the general population, psychiatric patients, or other offender groups. This result suggests that IED is prevalent among young offenders and deserves further scientific attention.

The relevance of psychopathic personality traits must also be mentioned. DeLisi et al. (2013), focusing on institutionalised delinquents, stated that rule-breaking behaviours appear early. The only significant predictor of total arrests and self-reported delinquency was the initiation of police contact or arrest. Outcomes were not predicted by either the onset of rule-breaking or the referral to juvenile court. In terms of psychopathic personality traits, only the onset of rule-breaking correlated with the level of psychopathy. Youths diagnosed with ADHD demonstrated a notably earlier onset of antisocial behaviour compared to those without the condition. Children with ADHD began violating rules 1.1 years ago. Youths with conduct disorder experienced an arrest significantly earlier than those without the disorder, having first been contacted by police 1.3 years prior and referred to juvenile court 0.8 years earlier. Children and adolescents with CD exhibited an earlier average onset across all three types, occurring 0.9 years sooner than those without CD. In another study, Kaplan and Cornell (2004) stated a weak relationship between psychopathy and ADHD. Indicators of ADHD had a negligible impact on the prediction of violent behaviour compared to the evaluation of juvenile psychopathy. ADHD itself did not increase the likelihood of violent institutional behaviour; however, along with the higher level of psychopathy, the chance of violent behaviour increased. In the research of Khanna et al. (2014), the Psychopathy Checklist’s interpersonal factor revealed significantly higher scores for the CD/ADHD group; this factor embodies the fundamental personality characteristics associated with psychopathy, such as grandiosity, manipulation, and pathological lying.

Substance use disorder (SUD) may also be a comorbid disorder among juvenile offenders. The follow-up study of Ståhlberg et al. (2017) aimed to examine institutionalised adolescents, categorising them into three groups: those with comorbid SUD and ADHD, those with SUD without ADHD, and those without SUD. Conducted over an average span of approximately three years post-institutionalisation, the study sought to assess rates of criminal behaviour, utilisation of inpatient healthcare services, and premature mortality and to investigate whether risk factors associated with group classification correlated with ongoing violent criminality. The overall results for these three groups were similar, marked by significant antisocial behaviours resulting in new sentences for many individuals, alongside a notable prevalence of illness. The SUD plus ADHD group reported a notably higher total of criminal acts charged in all convictions, with their numbers being twice as high as those observed in the non-SUD group. In this group, the inclination towards associating with delinquent peers for the planning and execution of crimes was less pronounced. Thus, their criminal activities relied less on being part of a similar peer group, as they predominantly committed offences independently. This suggests that individuals within the SUD plus ADHD group are more closely associated with an earlier initiation and a broader antisocial lifestyle, frequently engaging in criminal acts on their own rather than as members of a group of delinquent youths.

As we can see, several comorbid disorders may appear along with ADHD. However, it was not typical in the studies to discover a wide range of comorbid disorders and their potential impact on the behaviour of juvenile offenders. Sarver et al. (2014) investigated the relevance of conduct problems (ODD/CD symptoms) and substance use as mediators of risk concerning risky sexual behaviour and ADHD. They stated that mediation models indicated a direct link at first between symptoms of ADHD and self-reported sexual risk behaviour. This effect was explained by the separate pathways of problematic use of alcohol and marijuana, while conduct problems did not account for it. The connections between ADHD, substance use issues, and sexual risk behaviour (RSB) varied based on the existence of comorbid conduct problems in youths. In particular, the influence of ADHD on sexual risk behaviour was limited to a specific group of youths displaying notable comorbid conduct issues and was entirely mediated by problematic marijuana use. In contrast, there was no direct or indirect relationship between ADHD and RSB in youths who did not have heightened conduct problems. Overall, these findings suggest that the link between ADHD and RSB is indicative of the degree to which comorbid conduct issues, and especially problems related to substance use, have emerged.

Finally, the expenses of the offenders were measured only in the study of Jones et al. (2021), who found that, on average, service expenses for the ADHD group surpassed those of the non-ADHD group by USD 25,000 per child. For young individuals identified as having both ADHD and CD, the average costs for services reached over USD 80,000 across six years—more than double the expenditure for those with only ADHD and six times higher than that of a child without either disorder. Expenditures differ not just in their level but also in their composition. Further analysis of the data revealed that, when averaged over the years, there is a positive correlation between school service costs and juvenile justice costs for youth with ADHD, a pattern that does not appear in youth with CD or those with comorbid conditions. These statistics imply that for individuals with an ADHD diagnosis (but not CD), the requirement for support in school may indicate broader disciplinary issues that remain undiagnosed.

### 3.6. Academic Achievement

Of the studies reviewed, five were concerned with the educational attainment of young people. Their findings are in line with previous research suggesting that one of the main consequences of ADHD may be poorer academic outcomes as a result of inattention, overactivity, and impulsivity (Barkley, 2002; Fletcher & Wolfe, 2009).

The studies by Retz et al. (2004) and Rutten et al. (2022) showed that the educational attainment of the group of young people diagnosed with ADHD alone was lower than in Rutten’s study for young people with ASD (autism spectrum disorder) or ASD alone and in Retz’s study for young people without ADHD. The research by Margari et al. (2015) examined educational attainment by crime type, comparing property crimes, alcohol/drug-related crimes (54%), and crimes against people. It found that adolescents who committed property crimes (60%) and alcohol/drug-related crimes (54%) had higher rates of irregularity in their school careers than those who committed crimes against people. Poon and Suk-Han Ho (2015) investigated the reading ability of juvenile detainees in addition to ADHD. It found that juveniles with ADHD were associated with negative academic orientation; their difficulties with academic orientation were reflected in poor grades, less serious academic goals, and poorer planning. Interestingly, this is the only area where worse outcomes were reported for youth with ADHD compared to RD (reading disability); apart from this, it can be concluded that ADHD was associated with fewer psychosocial difficulties than RD.

In the study conducted by Atilola et al. (2017), the School Engagement Measure (SEM; Fredricks et al., 2004) was utilised to evaluate the engagement levels of respondents in school three months following their enrolment. The assessment covered all three aspects of school engagement: behavioural, emotional, and cognitive. Notably, the presence of a symptom cluster that met the DSM-5 diagnostic criteria correlated with significantly lower mean SEM scores across any of the three behavioural disorders examined among the respondents. Atilola’s research revealed that, on average, approximately two-thirds of the respondents had dropped out of school three years prior to their arrest or detention. Furthermore, there was an average gap of about four years between the anticipated duration of formal education at the time of arrest and detention. A clear link was identified between behavioural issues, particularly the symptom complex associated with ADHD, and increased dropout rates as well as lower educational outcomes for these individuals in comparison to their peers.

### 3.7. Methodology

Some studies used a diagnostic criteria system (DSM-IV, DSM-5, ICD-10) to identify ADHD, ODD (oppositional defiant disorder), CD (conduct disorder), or other comorbid disorders (Atilola et al., 2021; Chung et al., 2011; Margari et al., 2015; Rutten et al., 2022; Sarver et al., 2014; Sibley et al., 2011; Silva et al., 2014; Ståhlberg et al., 2017). In addition, diagnostic interviews (e.g., K-SADS) have been used in some cases (Khanna et al., 2014; Lindblad et al., 2020). However, several studies have used self-completion questionnaires to map behavioural and psychological characteristics, even for ADHD categorisation (Barra et al., 2022; Cho et al., 2013; Garg et al., 2024; Grieger & Hosser, 2012; Philipp-Wiegmann et al., 2018; Poon & Suk-Han Ho, 2015; Retz et al., 2004). In the case of some studies, the diagnostic criteria used were unclear, which determined the criteria used to assign offenders to ADHD in the study (Atilola et al., 2021; Kaplan & Cornell, 2004; Wojciechowski, 2021).

Several researchers (Grieger & Hosser, 2012; Silva et al., 2014) caution against establishing a clear link between mental health problems and offending. Silva et al. (2014) point out that the criminalisation of young people with mental health disorders has been raised as a concern by mental health professionals, advocacy groups, and researchers. Grieger and Hosser (2012) raise doubts along the recidivism line when they point out that the results of this study highlight the need for a theoretical and practical distinction between risk factors for delinquency and risk factors for criminal recidivism.

The significance of early intervention and crime prevention is underscored by a majority of studies (Atilola et al., 2021; Barra et al., 2022; Cho et al., 2013; Garg et al., 2024; Lindblad et al., 2020; Philipp-Wiegmann et al., 2018; Rösler et al., 2004; Sarver et al., 2014; Silva et al., 2014; Wojciechowski, 2021), which indicate that failing to address adolescent ADHD early on may lead to its progression into adult ADHD or the emergence of antisocial behaviours. More specific recommendations are provided by Barra et al. (2022), Silva et al. (2014), and Wojciechowski (2021), who emphasise the essential need for standardised yet individualised assessments of the risks and needs of young offenders or high-risk groups, conducted by professionals with psychiatric or psychological training (Barra et al., 2022). According to Silva et al. (2014), a comprehensive psychometric and mental health evaluation should be mandated for children entering the justice system, highlighting the necessity for thorough mental health screenings for all youth involved in the juvenile justice system (Wojciechowski, 2021).

Improvement in training health professionals operating within the juvenile justice system warrants attention (Silva et al., 2014). Practitioners in psychiatry, psychology, and law enforcement must collaborate with politicians and other stakeholders to develop and implement customised interventions for the effective screening and treatment of juveniles (Barra et al., 2022).

Opinions regarding treatment methods are, nonetheless, varied. According to research conducted by Garg et al. (2024), cognitive behavioural therapy, social skills training, and parent management training can effectively address symptoms of ADHD and ODD (obsessive–compulsive disorder). Conversely, another set of studies (Philipp-Wiegmann et al., 2018; Sarver et al., 2014; Sibley et al., 2011) suggest that a combination of medication and psychological interventions proves effective. They highlight that while pharmacological treatments are frequently employed for managing ADHD symptoms, relying solely on medication is unlikely to deter youth from engaging in RSB (Sarver et al., 2014). Furthermore, ADHD medication can lower criminality rates among forensic individuals with ADHD, aiming to prevent and disrupt maladaptive developmental trajectories (Philipp-Wiegmann et al., 2018).

## 4. Discussion

Because it is common for children with ADHD to be impulsive or hyperactive while trying to control their behaviour, which may increase the likelihood of developing conduct problems, the role of sociodemographic factors was under-represented in the studies examined. Regarding gender, most studies focused on male perpetrators and did not examine only female perpetrators. The results of gender comparisons showed that males were over-represented in the less severe ADHD subtype, while females were over-represented in the more severe ADHD subtype. Female offenders showed more severe ADHD, often in combination with other behavioural problems. This may suggest that ADHD symptoms in women tend to be more severe or complex, possibly with multiple co-occurring problems such as other behavioural disorders. This difference may contribute to different diagnostic and treatment practices between the sexes, as more severe forms in women may receive delayed or different diagnoses (Rösler et al., 2009; Young et al., 2020). However, adverse childhood experiences showed no significant difference. The prevalence and severity of ADHD may differ by gender, with adverse childhood experiences often associated with delinquent behaviour, having similar effects on both sexes (Arnett et al., 2015; Smallenburg et al., 2024). Another study found no significant difference in the age of first offending in boys and girls. Children diagnosed with ADHD, regardless of gender, were more likely to commit crimes. This confirms that ADHD is a strong risk factor for criminal behaviour and that there is a clear association between ADHD and juvenile delinquency for both males and females. Young people with ADHD may be more prone to impulsive, uncontrollable behaviours that can lead to crime (Ångström et al., 2024; Fletcher & Wolfe, 2009).

Sociodemographic factors and the role of the family have received attention in only a few studies. When examining the relationship between ADHD and violent behaviour, it can be seen that an ADHD diagnosis may be a significant contributor to chronic violent offending. Symptoms of ADHD, such as impulsivity, attention problems, and problems with emotion regulation, may contribute to increased violent behaviour, especially in the long term (Engelhardt et al., 2019; Retz & Rösler, 2010). The uncontrolled impulses and attention deficit often seen in ADHD can contribute to the repetitive nature of offending, particularly if the disorder is not adequately managed (Hamed et al., 2015). Race and socioeconomic status (SES) did not significantly affect the frequency of offending or different levels of offending. An ADHD diagnosis is an independent and stronger risk factor for criminal behaviour than traditionally important social factors. This may also suggest that although sociodemographic factors such as poverty, family background, or race generally influence criminality, the presence of ADHD may have an independent and independently outweighing effect on the commission of crime (Mordre et al., 2011; Pratt et al., 2002).

It can, therefore, be concluded that offenders with ADHD start to commit crimes at an earlier age (approximately one and a half to two years earlier) than offenders in the general population. This is in line with the literature, which consistently shows that ADHD is associated with earlier onset of disruptive and delinquent behaviour and contributes to life-persistent delinquency rather than to transient delinquency during adolescence (Loeber et al., 1995; Moffitt, 2003; Retz et al., 2004).

There are also inequalities in comorbidity, educational attainment, and living conditions among adolescents. Young people diagnosed with ADHD had lower educational attainment and family problems. Individuals with ADHD are often younger, have lower educational attainment, and face higher unemployment rates (Chronis-Tuscano & Bounoua, 2024; French et al., 2024). They also had a younger age at offending compared to young people without ADHD. The lower educational attainment and family problems of young people diagnosed with ADHD suggest that ADHD is not only a neurobiological condition but that social and environmental factors also contribute to their difficulties (Sibley et al., 2011; Zulauf et al., 2014). Symptoms of ADHD, such as impulsivity, attention problems, and organisational difficulties, can make school performance more difficult, leading to lower educational attainment later on (Colomer et al., 2017). Family problems, such as dysfunctional family environments or low-income families, can exacerbate this situation by not providing a stable background for improving school performance (Bussemakers et al., 2022; Kganyago Mphaphuli, 2023).

Psychosocial factors, such as age at first conviction, play an important role in the persistence of violent behaviour, and early conviction increases the risk of violent crime. Early conviction can be associated with negative labelling for young people, deepening the distance between society and the individual (Besemer et al., 2017). Labelling (stigma) can exacerbate criminal behaviour in the long term, as young people may internalise social rejection. If a young person enters the criminal justice system early, future rehabilitation, progression in the school system, or integration into the labour market may also be more difficult (Brehmer et al., 2024; Moore et al., 2016). Adverse childhood experiences also have a major impact on the behaviour of young offenders, as high levels of ACEs are present between the onset of intense offending and early offending. Children affected by ACEs often develop coping strategies, which may include aggressive patterns of behaviour to protect themselves or respond to threats (Engelhardt et al., 2019; Retz & Rösler, 2010). ACEs create traumatic experiences that often lead to later mental disorders (e.g., PTSD, anxiety, depression). These mental health problems are often associated with delinquent behaviour, as young people do not have adequate coping mechanisms to process their trauma (Elmore & Crouch, 2020; Gu et al., 2022).

Research shows that ADHD and its comorbidities have a significant impact on the behaviour and mental health of juvenile offenders. Several studies have shown that young people with ADHD often experience serious behavioural problems, including internalising (e.g., anxiety, depression) and externalising (e.g., aggression, conduct disorder) problems. Impulsivity and attention disorders may contribute to young people responding aggressively to situations (Saylor & Amann, 2016). Increased irritability, difficulty with emotion regulation, and sudden outbursts of anger are often present in people with ADHD (Connor et al., 2019). Young people with ADHD are often characterised by behavioural problems such as misbehaviour, resistance to authority, problems at school, or difficulties in social relationships. These disorders can be a precursor to criminal behaviour (Ferretti et al., 2019).

ADHD symptoms are often associated with other psychiatric disorders, such as impulsivity and social withdrawal. Individuals with ADHD are particularly prone to anxiety, depression, and impulsive behaviour (Knouse et al., 2013; Van Ameringen et al., 2011). ADHD is often associated with other mental disorders, such as depression, which is closely linked to juvenile delinquency. Young people with ADHD who have depression have lower self-esteem and more severe depressive symptoms (Mayer et al., 2022). In addition to depression, OCD and other psychiatric disorders are also common among individuals with ADHD, such as CD (conduct disorder), which increases the risk of delinquency (Gnanavel et al., 2019).

Among young offenders, behavioural problems such as oppositional defiant disorder are also common and have been strongly associated with ADHD. Research findings suggest that behavioural and social problems among juveniles with ADHD span a broad spectrum. Young people with ADHD are more likely to experience rejection from their peers, which has a long-term negative impact on their social relationships (Hoza, 2007). Inattentive behaviour means that young people with ADHD do not always follow subtle cues in social interactions, which can lead to misunderstandings (Carpenter Rich et al., 2009). Children and young people with ADHD often struggle to develop appropriate social skills. This includes regulating emotions, expressing empathy, and managing conflict constructively. Peers tend to avoid those who have difficulty fitting into social norms (Binti Marsus et al., 2022). Other disorders associated with ADHD, such as opposition defiant disorder (ODD) or conduct disorder (CD), exacerbate peer problems. Young people with these disorders often react more aggressively to conflict, which increases rejection (Ghosh & Sinha, 2012).

Although there are ambivalent results on crime and recidivism, some research suggests that offenders diagnosed with ADHD reoffend more quickly and commit more crimes than those without the disorder. Individuals with ADHD may be more likely to be in a society where rule-breaking behaviour is accepted or encouraged (Young & Cocallis, 2021). Individuals with impulsivity and adjustment problems may be more susceptible to the effects of such environments, which may maintain delinquent behaviour in the longer term (Chen & Jacobson, 2013). Additionally, individuals with ADHD do not always receive the psychological and psychiatric support they need in the criminal justice system. Without this, they find it more difficult to exit criminal behaviour and are more likely to reoffend (Anns et al., 2023; Freckelton, 2019).

Drug use is also a significant problem among young people with ADHD. The combination of ADHD and substance use disorder (SUD) is associated with a particularly high risk of delinquency. The co-occurrence of ADHD and SUD can contribute to increased delinquency, and such young people often have inadequate social relationships (Molina & Pelham, 2014). Attention deficit, impulsivity, and emotional instability may appear to be reduced in the short term by substance use, but this can quickly lead to addiction, which further impairs emotional and social functioning (Molina & Pelham, 2014).

Psychopathic personality traits also play a prominent role in the behaviour of offenders with ADHD. Psychopathic traits such as impulsivity, manipulation, and pathological lying are often present in young people with ADHD and other psychiatric disorders (Gray et al., 2019; Maurer et al., 2020). Research by DeLisi et al. (2013) highlighted that antisocial behaviour appears early in young people with ADHD and that antisocial personality traits can exacerbate criminal tendencies. Young people with ADHD and psychopathic traits have difficulty forming deep, emotionally based relationships, which can lead to isolation and a hostile worldview. This contributes to the perpetuation of antisocial behaviour and a complete lack of empathy when harming others (You et al., 2024).

The majority of juvenile ‘delinquents’ are children with psychosocial needs, often living on the streets and not attending school. This phenomenon is a feature of the developing world, as in most HICs, those caught for status and other minor offences are often diverted towards reintegration through non-prison alternatives (diversion programmes) and juvenile correctional forms of incarceration are primarily reserved for young people charged or convicted of more serious property or personal offences.

It can, therefore, be concluded that, in the long run, ADHD is associated with a decline in educational aspirations, with far-reaching negative consequences for socioeconomic outcomes in adulthood, and may also be a strong predictor of a greater propensity to commit crime (Rösler et al., 2004).

Overall, ADHD and its comorbidities have complex effects on adolescents’ behaviour and social inclusion. Behavioural problems associated with psychiatric disorders, such as aggression, anxiety, depression, and substance abuse, all contribute to increased risk of delinquency. Research emphasises that the treatment of ADHD should also take into account the presence of other comorbidities, as they can have a complex impact on young people’s lives.

From a methodological point of view, it should be noted that the results of self-completion questionnaires, diagnostic interviews, and structured scales have different validity and reliability, which may bias the results. In the case of self-completion questionnaires, the responses of individuals or caregivers may be influenced by memory biases, social conformity pressures, or the respondent’s own subjective interpretation. This can be particularly problematic for questions where we want to cluster patients along with behavioural problems. Self-completion questionnaires are not a substitute for structured interviews or clinical assessments, especially in diagnosing complex disorders such as ADHD or comorbidities. Clinical diagnostic measures should be used in research to increase reliability and validity. It should also be mentioned that in some studies, it was not clear what criteria were used to diagnose ADHD or other disorders, which reduces the validity of the data. Some studies have used strict diagnostic criteria, while others have used more flexible categorisation, which may distort prevalence data. Different sampling strategies (e.g., juvenile correctional, prison youth vs. school population) can significantly affect the prevalence and manifestation of ADHD and comorbid disorders. In addition, not all studies used a control group, making it difficult to generalise the results. In many cases, comorbid disorders (e.g., depression, PTSD, anxiety) have not been analysed in detail, which may bias the results and the understanding of the impact of ADHD. Socioeconomic factors, such as family background or school environment, have not received sufficient attention when interpreting the results. However, the consideration of these factors is of paramount importance, especially in the implementation of social reintegration and psychological support.

Although this review offered a detailed comparison between the studies included in the literature, the research is not without limitations. Limiting the review to English-language publications may have restricted the identification of relevant research. The exclusion of grey literature and non-English publications might have led to a publication bias, potentially omitting relevant data from under-represented regions or populations. Additionally, a substantial portion of the reviewed studies lacked control groups, limiting the ability to draw causal inferences. Furthermore, due to the variability and diversity of the included studies, it was not possible to calculate pooled sample sizes or effect sizes.

## 5. Conclusions

The above observations suggest that early identification and intervention are of paramount importance for young people with ADHD. Early diagnosis and appropriate treatment are essential to prevent delinquency. For young people with ADHD, there is a need for programmes that focus on addressing behavioural problems, developing social skills, and addressing mental health. Appropriate treatment and support programmes that target mental health, academic achievement, and social skills can help prevent later quality-of-life deterioration, unemployment, and delinquent behaviour. Strengthening the family environment and increasing support at school are also key to improving the situation. Psychological support (e.g., cognitive behavioural therapy, impulse control training, social skills development) and psychiatric treatments (e.g., medication for attention deficit disorder and comorbidities) can be effective in reducing the risk of crime.

Suggestions for solutions include the following:

- Targeted treatment and therapy: ADHD-specific psychotherapy programmes and medication can reduce impulsive behaviour.
- Skills development: Training in social and problem-solving skills can help improve social relationships and self-control.
- Structured environment: Building support systems that help people with ADHD live in a structured, predictable environment.
- Rehabilitation programmes: Specific programmes are needed in the justice system that consider ADHD-specific challenges.

## Data Availability

Not applicable.

## Appendix A

**Table A1:** Journals screened for papers.

Rank	Title	H Index	Total Docs. (3 Years)	Total Refs.	Total Cites (3 Years)	Citable Docs. (3 Years)	Cites/Doc. (2 Years)	Ref./Doc.	Country
1	*Journal of Behavioral Addictions*	88	294	3467	1865	293	5.75	40.31	Hungary
2	*Psychonomic Bulletin and Review*	187	524	18,115	2539	522	3.94	66.85	United States
3	*Journal of Memory and Language*	177	149	4006	578	145	3.45	80.12	United States
4	*International Journal of Clinical and Health Psychology*	59	148	7475	943	147	5.21	62.29	Spain
5	*Neuroscience of Consciousness*	20	88	2915	420	88	4.18	74.74	United Kingdom
6	*Comprehensive Psychiatry*	128	207	6728	1084	202	5.3	65.32	United States
7	*European Journal of Psychology Applied to Legal Context*	36	30	836	198	30	5.95	83.6	Spain
8	*Schizophrenia*	44	263	6989	1146	255	4	59.23	United Kingdom
9	*Australian Journal of Psychology*	56	100	1998	512	99	2.02	54	United States
10	*Social Cognitive and Affective Neuroscience*	132	319	6922	1319	316	3.34	72.1	United Kingdom
11	*Psychosocial Intervention*	35	49	1596	269	47	5.63	84	Spain
12	*Annual Review of Applied Linguistics*	68	38	774	201	36	6.1	77.4	United Kingdom
13	*Educational Psychology*	90	220	3591	923	198	3.41	61.91	United Kingdom
14	*Collabra: Psychology*	30	268	7295	989	268	3.7	72.23	United States
15	*Cognitive Research: Principles and Implications*	45	246	4666	1022	243	3.44	58.33	United Kingdom
16	*Borderline Personality Disorder and Emotion Dysregulation*	37	100	1809	400	98	2.87	58.35	United Kingdom
17	*Journal of Criminal Justice*	106	246	9920	1083	245	3.15	71.37	United Kingdom
18	*Psicothema*	85	181	2444	689	180	3.74	58.19	Spain
19	*Addiction Science and Clinical Practice*	51	213	4982	679	202	2.83	50.84	United Kingdom
20	*Psychologica Belgica*	41	60	947	214	59	3.34	63.13	United Kingdom
21	*Nature and Science of Sleep*	55	457	8817	1690	448	3.63	48.18	New Zealand
22	*Autism and Developmental Language Impairments*	22	73	1804	320	73	3.83	72.16	United Kingdom
23	*Research in Psychotherapy: Psychopathology, Process and Outcome*	21	95	1378	258	93	3.24	57.42	Italy
24	*Judgment and Decision Making*	78	151	2032	378	148	2.17	56.44	United States
25	*Frontiers in Behavioral Neuroscience*	106	1226	10,522	3427	1110	2.57	64.55	Switzerland
26	*Eating and Weight Disorders*	66	751	4217	2398	715	2.96	55.49	Switzerland
27	*Journal of Intelligence*	32	408	8818	1607	399	3.81	67.83	Switzerland
28	*BioPsychoSocial Medicine*	43	91	976	268	87	2.18	42.43	United Kingdom
29	*International Review of Social Psychology*	30	63	1464	180	63	1.86	69.71	United Kingdom
30	*Revista de Psicodidactica*	38	60	1194	258	60	4.2	59.7	Spain

**Table A2:** The characteristics of the papers analysed.

	Location, Period	Sample	Diagnosis of ADHD	Comorbid Disorder	Special Aspect	Aim	Tool	Treatment, Suggestion
1. Kaplan and Cornell (2004)	Diagnostic Center of the Virginia Department of Juvenile Justice	N = 122 males age: 13–18 years 25% with clinical diagnosis of ADHD	yes (not reported based on what)	24% had a dual diagnosis of ADHD and conduct disorder 6%: ADHD + oppositional defiant disorder	psychopathy and violent	How are psychopathy and ADHD related? In what ways does ADHD affect the connection between psychopathy and violent behaviour?	Psychopathy Checklist Revised (PCL-R). Diagnosis of ADHD. History of psychostimulant medication. The ADHD scale of the Personality Inventory for Youth (PIY).	Clinicians working within the juvenile justice system must be careful when asserting a causal connection between psychopathy and ADHD, as they have identified overlapping symptoms, yet no substantial relationship exists.
2. Barra et al. (2022)	A juvenile detention centre in Worms, Germany, between May 2018 and May 2019	N = 156 (129 male, 82.7%; 27 female, 17.3%) age: 14–25 years	no (self-reported, self-report Wender–Reimherr adult attention deficit disorder scale)		ADHD, IED, ACEs, and further psychiatric/psychological impairments	To obtain advanced understanding of the prevalence and connections between ADHD, IED, ACEs, and other psychiatric or psychological disorders in young offenders, both male and female.	Self-report Wender–Reimherr adult attention deficit disorder scale (SR-WRAADDS). Intermittent explosive disorder screening questionnaire for DSM-5 (IED-SQ). Childhood trauma questionnaire—short form (CTQ-SF). Youth self-report (YSR).	The necessity for early detection of ACEs and ADHD/IED among young offenders is underscored by the findings, aiming to pinpoint adolescents who face a heightened risk of enduring criminal careers. Collaboration among practitioners in psychiatry, psychology, law enforcement, politicians, and other stakeholders is essential for the development and execution of customised interventions.
3. Khanna et al. (2014)	2010–2011 in custody in the northwest region of England	N = 109 male White British age: 12–18 years	yes (DSM, K-SADS)	N = 76 with CD N = 33 with CD/ADHD	CD In the year following their release from custody, 36 young individuals were found guilty of at least one violent crime.	The aim is to evaluate the predictive accuracy of the SAVRY, YLS/CMI, and PCL:YV among juvenile offenders diagnosed with conduct disorder, both with and without ADHD. It is hypothesised that those with co-occurring ADHD will score higher on risk assessment instruments and that these individuals will be more prone to reoffending within a year of their release from custody.	The Structured Assessment of Violence Risk in Youth (SAVRY). The Psychopathy Checklist: Youth Version (PCL:YV). The Youth Level of Service/Case Management Inventory (YLS/CMI). The K-SADS was used to ensure that none of the participants had an undiagnosed current major mental illness and assessed participants for ADHD.	
4. Poon and Suk-Han Ho (2015)	Hong Kong	N = 117 boys age: 12.42–18.17 years divided into four groups: AS (*n* = 29), RD (*n* = 24), AS + RD (*n* = 35), and control (*n* = 29)	no (AS was examined instead of full-structured diagnostic ADHD)	reading disability (RD)		The primary aim is to investigate the psychosocial effects on a juvenile sample exhibiting symptoms of ADHD (AS), RD, and their co-occurrence across various psychosocial areas. The secondary aim is to assess the influence of comorbidity by analysing the severity of delinquency across all groups.	General intellectual ability. Hong Kong Wechsler Intelligence Scale for Children (HK-WISC). Hong Kong Test of Specific Learning Difficulties in Reading and Writing for Junior Secondary School Students (HKT-JS). TRF Attention Problems scale.	
5. Lindblad et al. (2020)	Russia	N = 370 adolescents 98% Caucasian age: 14–19 years the majority of participants came from economically disadvantaged backgrounds, often having unemployed parents, characterised by low educational attainment	yes (K-SADS-PL)	CD N = 65 (17.6%) ADHD diagnosis N = 271 (73.2%) CD diagnosis	Prevalence of comorbid diagnoses aggression, impulsiveness and alcohol-related problems in delinquents with psychopathic traits	This study aimed to explore the relationships between comorbidity, aggression, impulsivity, and psychopathic characteristics in young offenders diagnosed with both ADHD and CD.	Psychopathology was assessed through a semi-structured psychiatric interview. A subsample of more than 300 participants also completed a set of self-reports. Combined psychiatric diagnoses. The Schedule for Affective Disorders and Schizophrenia for School-Age Children– Present and Lifetime Version (K-SADS-PL). Aggression Questionnaire (AQ). Antisocial Behavior Checklist (ABC). Barratt Impulsiveness Scale (BIS-11). Rutgers Alcohol Problem Index (RAPI). Antisocial Process Screening Device (APSD). Childhood Psychopathy Scale (CPS).	The significant occurrence of PTSD linked to ADHD/CD delivers a crucial clinical insight by emphasising the heightened risk of traumatic experiences within this population. The results clearly indicate the necessity for early intervention and diligent oversight when both ADHD and CD are present.
6. Cho et al. (2013)	South Korea	N = 275 age: 13–18 years 32.4% with ADHD 52.0% with depression	no (self-reported, ADHD DSM from youth self-report)		Identify factors that influence depression in juvenile offenders.	To identify factors that influence depression in juvenile offenders.	The ADHD symptoms scale and the ADHD DSM form youth self-report (YSR). Self-esteem by Rosenberg and revised by Jeong. The instrument used to measure depression was the Center for Epidemiological Studies Depression Scale (CES-D).	Adolescents with ADHD may experience significant learning difficulties, troubled interactions with peers or educators, lowered self-esteem, and heightened levels of anxiety and depression, which can lead to an increased risk of delinquent behaviour. Consequently, proactive intervention is essential, as failing to address adolescent ADHD early on could result in its progression into adult ADHD or antisocial conduct.
7. Rutten et al. (2022)	Netherlands 2013–2014	N = 188 from a compilation of all pre-trial forensic psychiatric and psychological evaluations of male adolescents aged 12 to 17 in the Netherlands for the years 2013 and 2014	yes (had been diagnosed based on the pre-trial forensic assessment report)	ASD out of the 1799 pre-trial evaluations conducted on these male adolescents, 69 were diagnosed with ASD, 90 with ADHD, and 29 received diagnoses for both conditions	ASD	The objective was to examine if the nature of reported index offences among 12-to-17-year-olds varies among those diagnosed with ASD, ADHD, or a combination of both ASD and ADHD.	A checklist of 76 items that includes characteristics related to the following: Health and criminal behaviour. Psychiatric diagnoses, educational attainment, living circumstances, and medication usage at the time of the alleged index offence. The highest level of graduation achieved is indicated by the education level. Characteristics related to both the index offence, past offences, convictions, and recommendations for treatment from psychiatrists or psychologists.	
8. Garg et al. (2024)	India	N = 60 male juvenile offenders between the ages of 12 and 17	no (Child Behavioural Checklist—CBCL)	ODD (oppositional defiant disorder)		This study aims to investigate the relationship between behaviour issues in juvenile offenders and oppositional defiant disorder (ODD) and attention deficit/hyperactivity disorder (ADHD). The study’s objective is to assess the incidence of ADHD and ODD symptoms among juvenile offenders and how they relate to the emergence of behavioural issues.	Child Behaviour Checklist (CBCL).	There is a need for the early identification and intervention of ADHD and ODD symptoms in children who are at risk for behavioural challenges and criminal conduct. By tackling ADHD and ODD from the outset, professionals can strive to diminish the chances of conduct-related issues and foster positive results for young offenders. The presence of both ADHD and ODD can greatly affect an individual’s functioning, social interactions, and academic performance. Symptoms associated with ADHD and ODD can be effectively addressed through cognitive behavioural therapy, social skills training, or parent management training.
9. Philipp-Wiegmann et al. (2018)	2001–2016 Germany	2001: N = 129 2016: criminal record screening in 2016 (N = 108)	no (Wender Utah Rating Scale (WURS-k))		15-year follow-up study. According to the results of the WURS-K and the ADHD-DC, subjects were allocated to two overlapping groups, Lifetime ADHD and Adult ADHD.	The influence of ADHD on the criminal trajectories of young men in prison was examined. To assess the likelihood of relapse among individuals with and without ADHD, survival analyses were conducted.	German short version of the Wender Utah Rating Scale (WURS-k). ADHD diagnostic checklist (ADHD-DC). International Personality Disorder Examination (IPDE). DSM-IV criteria using the structured clinical interview SCID-I. HAWIE-R. Recidivism.	There is a need for sufficient ADHD interventions not only within the general population but also among forensic groups, highlighting the importance of an early intervention approach aimed at prevention to disrupt maladaptive developmental trajectories.
10. Retz et al. (2004)	Saarland/Germany	N = 129 young male delinquents age: 15–28 years M = 19.5 years (SD 2.0)	no			To detail the occurrence of ADHD and associated disorders among young adult inmates in juvenile detention, based on DSM-IV and ICD-10 standards.	Wender Utah Rating Scale—German short version (WURS-k). ADHD Diagnostic Checklist (ADHD-DC). The Wender–Reimherr Interview. The NEO Five-Factor Inventory (NEO-FFI). The youth self-report (YSR) and the young adult self-report (YASR).	The identification of ADHD in adults may benefit from the application of psychometric tools. Furthermore, it emphasises the importance of early diagnosis and treatment to deter children with ADHD from embarking on a path toward criminal behaviour. There are sufficient treatment options available for adult ADHD, and specialised programmes in juvenile correctional facilities could potentially reduce the likelihood of reoffending among young offenders with ADHD.
11. Ståhlberg et al. (2017)	Sweeden 2004–2007	N = 100 age: 12–19 3 subgroups: 1. with comorbid substance use disorders (SUDs) and ADHD (*n* = 25) 2. only SUD but no ADHD (*n* = 30) 3. without SUD (*n* = 45)	yes (DSM)		3-year follow-up study Focus: ADHD and substance use disorders (SUDs)	To analyse the trends of violent and overall criminal behaviour, both prior to and during follow-up, among adolescents placed in youth institutional care with (i) co-occurring SUD and ADHD, (ii) SUD alone without ADHD, and (iii) no SUD. To evaluate the frequency of inpatient healthcare visits and premature deaths across these three categories.	Background information and demographic details encompass (i) comprehensive data on academic performance highlighting various types of school-related issues, (ii) records of criminal activity, (iii) instances of substance use detailing specific drugs, and (iv) family dynamics. Wechsler Adult Intelligence Scale (WAIS) and Wechsler Intelligence Scale for Children (WISC). DSM-IV-based diagnostics. SUD according to DSM-IV criteria.	This indicates that merely institutionalising adolescents exhibiting externalising and delinquent behaviours is inadequate as an intervention for preventing their criminal activities. The age at which an individual receives their first conviction serves as a fairly reliable indicator of continued engagement in violent crime. This discovery highlights the critical need to recognise the early signs of criminal behaviour, which serve as a major indicator of a heightened risk for developing ongoing patterns of aggressive and antisocial reactions and behaviours.
12. Sibley et al. (2011)		N = 288 males with childhood ADHD and N = 209 demographically similar males without ADHD age: 5.0–12.83 years (M = 8.92, SD = 1.79)	yes (DSM)	CD		The research investigated the link between childhood ADHD and juvenile delinquency by analysing data from the Pittsburgh ADHD Longitudinal Study (PALS), which followed individuals diagnosed with ADHD during childhood (ages 5–12) and reconnected with them in their adolescence and young adulthood for annual assessments.	PALS follow-up interviews were conducted yearly beginning in the year of enrolment. Self-reported delinquency questionnaire (SRD)	Future studies need to explore factors like parenting that take place between childhood and adolescence, as these likely affect a child with ADHD’s likelihood of starting or continuing down an antisocial path. Moreover, upcoming research should investigate the continuation of criminal behaviour into adulthood among individuals with ADHD, thereby broadening the patterns analysed in the present study and determining if the early-onset offences observed in the ADHD cohort signify a trajectory of life-course-persistent criminality.
13. Grieger and Hosser (2012)	Germany	N = 283 age: 15–24 years (M = 19.0, SD = 1.9)	no (FEA-FSB and FEA-ASB)		5 years follow-up period The interviews were conducted at the start of a prisoner’s sentence (after an average of 2.4 months in prison, SD = 4.3), five months into their incarceration (M = 5.0, SD = 4.0), and just prior to their anticipated release (M = 0.4 months, SD = 2.3).	The study aimed to determine if ADHD serves as a predictor for recidivism. It was assumed that ADHD constitutes a risk factor for recidivism, which is a specific instance of delinquent behaviour. Additionally, it was believed that a diagnosis of ADHD might be associated with an earlier return to offending following release. They anticipated that both assumptions would also hold true in instances of violent reoffending.	Trained interviewers (N = 13) in standardised, oral interviews. Fragebogen zur Erfassung von ADHS im Erwachsenenalter. Intelligence quotient (IQ), substance dependence, and conduct disorder. Information on recidivism was obtained from the German Federal Central Criminal Register. SKID-II interview. Control variables from government records.	The findings of this study underscore the importance of differentiating between risk factors associated with delinquency and those linked to criminal recidivism in both theory and practice. While ADHD may be identified as a risk factor for delinquency, it does not serve as a predictor for recidivism.
14. Silva et al. (2014)	Between January 1995 and December 2010 in western Australia	N = 12,831 non-Indigenous Australian age: 10–21 years, diagnosed and treated with stimulant drugs for ADHD N = 792 (8%) boys and N = 75 (3%) girls with ADHD N = 822 (4%) boys and N = 75 (1%) girls without ADHD	yes (DSMIV/ICD10)		population-based cohort study	Attention deficit/hyperactivity disorder (ADHD) stands as the most common neurodevelopmental disorder among children and is occasionally observed in young individuals and adults retrospectively, particularly those who are incarcerated. Our objective was to explore encounters with the juvenile justice system involving children both with and without ADHD.	DSMIV/ICD10 criteria for ADHD and have symptoms severe need for drugs. Community correction records and incarceration records. Demographic information on sex, maternal marital status, maternal age at birth of the child (30 years), and gestation.	The health and social service requirements of every juvenile entering the justice system must be evaluated, and ongoing assistance and support should be offered upon their reintegration into the community. Efforts should focus on decreasing reoffending through improved support and management strategies during detention and after release. Ultimately, there is a need to enhance the training of health professionals operating within the juvenile justice system.
15. Wojciechowski (2021)	2000–2003 in Maricopa County (Arizona) and Philadelphia (Pennsylvania)	The Pathways to Desistance data N = 1354 juvenile offenders for 84 months following conviction for a serious offence all participants were between ages 14–18 at baseline	yes but not specified			This study investigates ADHD as a potential risk factor for predicting group trajectory assignments.	Computer-assisted interview (CAI) technology. Violent offending—self-reported engagement. Control variables: gender, race, SES.	The findings of this research indicate that individuals who meet the diagnostic criteria for ADHD are also more likely to exhibit higher levels of violent behaviour. This underscores the importance of thorough mental health assessments for all young people entering the juvenile justice system, as such evaluations could reveal previously undetected mental health concerns.
16. Atilola et al. (2017)	2020 Youth Correctional Centre for Boys, Oregun, Lagos, Nigeria	N = 103 adolescent boys between the ages of 12 and 17 years		Among the respondents, persistent behavioural disorders were prevalent, with as many as 54%, 39%, and 26% respectively fulfilling the DSM-5 criteria for conduct disorder, oppositional defiant disorder, and ADHD.		The current research highlights possible obstacles to comprehensive rehabilitation, particularly regarding educational re-engagement, for youth involved in the justice system in Africa. This was achieved through the analysis of quantitative data addressing the educational and psychosocial challenges faced by a group of justice-involved youth, alongside qualitative insights into the actual conditions within a correctional school located in a youth correctional facility in Lagos.	Basic social and demographic characteristics. Kiddie Schedule for Affective Disorders and Schizophrenia. School Engagement Measure.	A crucial advancement in reshaping Africa’s juvenile justice system into a genuinely rehabilitative and reformatory environment for troubled and offending youth is the establishment of diversion programmes across the region. In correctional schools, the resources available fall short of facilitating effective learning, leading to prevalent behavioural issues that often go unnoticed, which in turn adversely affects school engagement after students are enrolled. There is a necessity to officially integrate psychosocial and educational assessments prior to intake, along with intervention plans after identification, into the functioning of juvenile justice facilities across Africa.
17. Sarver et al. (2014)	2012 southeastern United States	ethnically diverse cross-sectional sample of adolescents (N = 115; mean age = 14.9 years) 84% male and 16% female			randomised clinical trial (RCT) The criteria for inclusion in the RCT are as follows: (a) ages 12 to 17, (b) either formal or informal probationary status, and (c) fluency in English for both the youth and their parent/caregiver.	Not specified	Diagnostic Interview Schedule for Children-IV, 4.0 Present State version (DISC-IV). Self-reported substance use. RSB—The Sexual Risk Behavior Scale (SRBS). Recruitment site, youth age, gender, race/ethnicity, and annual household income.	Timely recognition and management of these associated conditions could be crucial in preventing adverse sexual health effects in young people with ADHD. Although medication is frequently prescribed to address ADHD symptoms, it is improbable that it will solely stop youth from participating in risky sexual behaviour. Consistent evaluation for behavioural issues, substance abuse, and risky sexual behaviours appears necessary for adolescents undergoing ADHD treatment.
18 Jones et al. (2021)	Durham, NC; Nashville, TN; Seattle, WA; and central Pennsylvania, USA	N = 650 children in the high-risk group	not specified		cost	The patterns of service utilisation and expenses for adolescents diagnosed with attention deficit/hyperactivity disorder (ADHD) along with comorbid conduct disorder (CD) were evaluated during the ages of 12 to 17. The analysis focused on key service sectors such as mental health, educational services, and the juvenile justice system.	Data are provided by three cohorts from the Fast Track evaluation and are based on parent report. Diagnostic groups are identified through a structured assessment.	
19. Margari et al. (2015)	University of Bari Aldo Moro	N = 135 juvenile offenders age: 14–18 years	not specified			The purpose of this research was to assess possible environmental and psychological risk factors, with particular emphasis on ADHD symptoms, among a group of young offenders based on the nature of their offences.	Youth self-report (YSR) for ages 11–18. Conners’ Adolescent Self-Report Scale (CASS). Semistructured interview. Criminal record. Quality of family relationships. Peer relationships. Academic achievement. Antisocial behaviour.	
20 Chung et al. (2011)	2015 Korea	N = 251 N = 149 from the juvenile delinquency group and N = 102 from the comparison group	not specified		This study aims to assess the prevalence of externalising symptoms like ADHD and internalising symptoms such as depression, anxiety, suicidal thoughts, self-esteem issues, and alcohol problems among juvenile delinquents in Korea, marking the first evaluation of its kind in the country.	This research aims to assess the prevalence of externalising symptoms like ADHD, along with internalising symptoms such as depression, anxiety, suicidal thoughts, self-esteem issues, and alcohol-related problems among juvenile delinquents in Korea for the first time.	Epidemiological questionnaire. Korean ADHD rating scales (KARS). Beck scale for suicidal ideation. Beck’s depression inventory. Beck’s anxiety inventory. Rosenberg’s self-esteem inventory. Cut-down, annoyed, guilty-feeling, eye-opening alcohol questionnaire.	To prevent juvenile delinquency, it is essential to give particular focus and care to teenagers who exhibit high levels of ADHD or struggle with low self-esteem.
21 DeLisi et al. (2013)	Pennsylvania June–August 2009	N = 252 boys and girls age: 14–18 years having been in the facility between 3 and 12 months when recruitment started (N = 152)	not specified			This study aimed to clarify the relationship between the onset of crime and three indicators of antisocial behaviour: the initiation of rule-breaking or law violations, the beginning of police interactions or arrests, and the first referral to juvenile court. It also examined how these factors relate to different antisocial outcomes in a group of institutionalised juvenile delinquents from private residential facilities in Pennsylvania.	Structured one-on-one interviews using computer-assisted survey interview (CASI) techniques. Self-reported delinquency. Youth Psychopathic Inventory (YPI). ADHD diagnosis or CD diagnosis. Control variables (sex, age, and race to offending careers and antisociality).	

## References

[B1-behavsci-15-01044] Anns F., D’Souza S., MacCormick C., Mirfin-Veitch B., Clasby B., Hughes N., Forster W., Tuisaula E., Bowden N. (2023). Risk of criminal justice system interactions in young adults with attention-deficit/hyperactivity disorder: Findings from a national birth cohort. Journal of Attention Disorders.

[B2-behavsci-15-01044] Arnett A. B., Pennington B. F., Willcutt E. G., DeFries J. C., Olson R. K. (2015). Sex differences in ADHD symptom severity. Journal of Child Psychology and Psychiatry, and Allied Disciplines.

[B3-behavsci-15-01044] Atilola O., Abiri G., Adebanjo E., Ola B. (2021). The cross-cutting psychosocial and systemic barriers to holistic rehabilitation, including educational re-engagement, of incarcerated adolescents: Realities in and perspectives from Africa. International Journal of Educational Development.

[B4-behavsci-15-01044] Atilola O., Ola B., Abiri G., Sahid-Adebambo M., Odukoya O., Adewuya A., Coker O., Folarin O., Fasawe A. (2017). Status of mental-health services for adolescents with psychiatric morbidity in youth correctional institutions in Lagos *. Journal of Child & Adolescent Mental Health.

[B5-behavsci-15-01044] Ångström A., Andersson A., Garcia-Argibay M., Chang Z., Lichtenstein P., D’Onofrio B. M., Tuvblad C., Ghirardi L., Larsson H. (2024). Criminal convictions in males and females diagnosed with attention deficit hyperactivity disorder: A Swedish national registry study. JCPP Advances.

[B6-behavsci-15-01044] Babinski L. M., Hartsough C. S., Lambert N. M. (1999). Childhood conduct problems, hyperactivity-impulsivity, and inattention as predictors of adult criminal activity. Journal of Child Psychology and Psychiatry, and Allied Disciplines.

[B7-behavsci-15-01044] Baggio S., Fructuoso A., Guimaraes M., Fois E., Golay D., Heller P., Perroud N., Aubry C., Young S., Delessert D., Gétaz L., Tran N. T., Wolff H. (2018). Prevalence of attention deficit hyperactivity disorder in detention settings: A systematic review and meta-analysis. Frontiers in Psychiatry.

[B8-behavsci-15-01044] Barker T. H., Habibi N., Aromataris E., Stone J. C., Leonardi-Bee J., Sears K., Hasanoff S., Klugar M., Tufanaru C., Moola S., Munn Z. (2024). The revised JBI critical appraisal tool for the assessment of risk of bias for quasi-experimental studies. JBI Evidence Synthesis.

[B9-behavsci-15-01044] Barkley R. A. (2002). Major life activity and health outcomes associated with attention-deficit/hyperactivity disorder. The Journal of Clinical Psychiatry.

[B10-behavsci-15-01044] Barkley R. A., Fischer M., Smallish L., Fletcher K. (2004). Young adult follow-up of hyperactive children: Antisocial activities and drug use. Journal of Child Psychology and Psychiatry.

[B11-behavsci-15-01044] Barra S., Turner D., Müller M., Hertz P. G., Retz-Junginger P., Tüscher O., Huss M., Retz W. (2022). ADHD symptom profiles, intermittent explosive disorder, adverse childhood experiences, and internalizing/externalizing problems in young offenders *. European Archives of Psychiatry and Clinical Neuroscience.

[B12-behavsci-15-01044] Barzman D. H., Fieler L., Sallee F. R. (2004). Attention-deficit hyperactivity disorder diagnosis and treatment. Separating myth from substance. The Journal of Legal Medicine.

[B13-behavsci-15-01044] Besemer S., Farrington D. P., Bijleveld C. C. J. H. (2017). Labeling and intergenerational transmission of crime: The interaction between criminal justice intervention and a convicted parent. PLoS ONE.

[B14-behavsci-15-01044] Biederman J., Faraone S. V. (2005). Attention-deficit hyperactivity disorder. The Lancet.

[B15-behavsci-15-01044] Binti Marsus N., Sook Huey L., Saffari N., Motevalli S. (2022). Peer relationship difficulties among children with adhd: A systematic review. International Journal of Academic Research in Business and Social Sciences.

[B16-behavsci-15-01044] Brehmer C. E., Qin S., Young B. C., Strauser D. R. (2024). Self-stigma of incarceration and its impact on health and community integration. Criminal Behaviour and Mental Health.

[B17-behavsci-15-01044] Bussemakers C., Kraaykamp G., Schoon I., Tolsma J. (2022). Household dysfunction and child development: Do financial resources matter?. Advances in Life Course Research.

[B18-behavsci-15-01044] Carpenter Rich E., Loo S. K., Yang M., Dang J., Smalley S. L. (2009). Social functioning difficulties in ADHD: Association with PDD risk. Clinical Child Psychology and Psychiatry.

[B19-behavsci-15-01044] Chae P. K., Jung H. O., Noh K. S. (2001). Attention deficit hyperactivty disorder in Korean juvenile delinquents. Adolescence.

[B20-behavsci-15-01044] Chen P., Jacobson K. C. (2013). Impulsivity moderates promotive environmental influences on adolescent delinquency: A comparison across family, school, and neighborhood contexts. Journal of Abnormal Child Psychology.

[B21-behavsci-15-01044] Cho Y., Gang M., Oh K. (2013). ADHD symptoms, self-esteem, and depression of juvenile offenders *. Journal of Digital Convergence.

[B22-behavsci-15-01044] Chronis-Tuscano A., Bounoua N. (2024). ADHD prevalence rose, yet disparities remain: Commentary on the 2022 national survey of children’s health. Journal of Clinical Child & Adolescent Psychology.

[B23-behavsci-15-01044] Chung H. L., Mulvey E. P., Steinberg L. (2011). Understanding the school outcomes of juvenile offenders: An exploration of neighborhood influences and motivational resources *. Journal of Youth and Adolescence.

[B24-behavsci-15-01044] Colomer C., Berenguer C., Roselló B., Baixauli I., Miranda A. (2017). The impact of inattention, hyperactivity/impulsivity symptoms, and executive functions on learning behaviors of children with ADHD. Frontiers in Psychology.

[B25-behavsci-15-01044] Connor D. F., Newcorn J. H., Saylor K. E., Amann B. H., Scahill L., Robb A. S., Jensen P. S., Vitiello B., Findling R. L., Buitelaar J. K. (2019). Maladaptive aggression: With a focus on impulsive aggression in children and adolescents. Journal of Child and Adolescent Psychopharmacology.

[B26-behavsci-15-01044] Coolidge F. L., Thede L. L., Young S. E. (2000). Heritability and the comorbidity of attention deficit hyperactivity disorder with behavioral disorders and executive function deficits: A preliminary investigation. Developmental Neuropsychology.

[B27-behavsci-15-01044] Cornish K. M., Manly T., Savage R., Swanson J., Morisano D., Butler N., Grant C., Cross G., Bentley L., Hollis C. P. (2005). Association of the dopamine transporter (DAT1) 10/10-repeat genotype with ADHD symptoms and response inhibition in a general population sample. Molecular Psychiatry.

[B28-behavsci-15-01044] DeLisi M., Neppl T. K., Lohman B. J., Vaughn M. G., Shook J. J. (2013). Early starters: Which type of criminal onset matters most for delinquent careers? *. Journal of Criminal Justice.

[B29-behavsci-15-01044] De Sanctis V. A., Nomura Y., Newcorn J. H., Halperin J. M. (2012). Childhood maltreatment and conduct disorder: Independent predictors of criminal outcomes in ADHD youth. Child Abuse & Neglect.

[B30-behavsci-15-01044] Eaves L., Rutter M., Silberg J. L., Shillady L., Maes H., Pickles A. (2000). Genetic and environmental causes of covariation in interview assessments of disruptive behavior in child and adolescent twins. Behavior Genetics.

[B31-behavsci-15-01044] Elmore A. L., Crouch E. (2020). The association of adverse childhood experiences with anxiety and depression for children and youth, 8 to 17 years of age. Academic Pediatrics.

[B32-behavsci-15-01044] Engelhardt P. E., Nobes G., Pischedda S. (2019). The relationship between adult symptoms of attention-deficit/hyperactivity disorder and criminogenic cognitions. Brain Sciences.

[B33-behavsci-15-01044] Faraone S. V., Biederman J., Monuteaux M. C. (2000). Toward guidelines for pedigree selection in genetic studies of attention deficit hyperactivity disorder. Genetic Epidemiology.

[B34-behavsci-15-01044] Farrington D. P. (2003). Developmental and life-course criminology: Key theoretical and empirical issues-the 2002 sutherland award address. Criminology.

[B35-behavsci-15-01044] Farrington D. P. (2016). Family influences on offending and family-based intervention. Women and children as victims and offenders: Background, prevention, reintegration: Suggestions for succeeding generations, Vol. I.

[B36-behavsci-15-01044] Ferretti N. M., King S. L., Hilton D. C., Rondon A. T., Jarrett M. A. (2019). Social functioning in youth with attention-deficit/hyperactivity disorder and sluggish cognitive tempo. The Yale Journal of Biology and Medicine.

[B37-behavsci-15-01044] Fletcher J., Wolfe B. (2009). Long-term consequences of childhood ADHD on criminal activities. The Journal of Mental Health Policy and Economics.

[B38-behavsci-15-01044] Foley H. A., Carlton C. O., Howell R. J. (1996). The relationship of attention deficit hyperactivity disorder and conduct disorder to juvenile delinquency: Legal implications. Bulletin of the American Academy of Psychiatry & the Law.

[B39-behavsci-15-01044] Forehand R., Wierson M., Frame C., Kempton T., Armistead L. (1991). Juvenile delinquency entry and persistence: Do attention problems contribute to conduct problems?. Journal of Behavior Therapy and Experimental Psychiatry.

[B40-behavsci-15-01044] Freckelton I. (2019). Attention deficit hyperactivity disorder (ADHD) and the criminal law. Psychiatry, Psychology and Law.

[B41-behavsci-15-01044] Fredricks J. A., Blumenfeld P. C., Paris A. H. (2004). School engagement: Potential of the concept, state of the evidence. Review of Educational Research.

[B42-behavsci-15-01044] French B., Nalbant G., Wright H., Sayal K., Daley D., Groom M. J., Cassidy S., Hall C. L. (2024). The impacts associated with having ADHD: An umbrella review. Frontiers in Psychiatry.

[B43-behavsci-15-01044] Garg P., Sharma G., Sharma H., Acharya C. (2024). Impact of ODD and ADHD on conduct problems among juvenile delinquents *. Universal Journal of Public Health.

[B44-behavsci-15-01044] Ghosh S., Sinha M. (2012). ADHD, ODD, and CD: Do they belong to a common psychopathological spectrum? A case series. Case Reports in Psychiatry.

[B45-behavsci-15-01044] Gnanavel S., Sharma P., Kaushal P., Hussain S. (2019). Attention deficit hyperactivity disorder and comorbidity: A review of literature. World Journal of Clinical Cases.

[B46-behavsci-15-01044] Gray N. S., Weidacker K., Snowden R. J. (2019). Psychopathy and impulsivity: The relationship of psychopathy to different aspects of UPPS-P impulsivity. Psychiatry Research.

[B47-behavsci-15-01044] Grieger L., Hosser D. (2012). Attention deficit hyperactivity disorder does not predict criminal recidivism in young adult offenders: Results from a prospective study *. International Journal of Law and Psychiatry.

[B48-behavsci-15-01044] Gu W., Zhao Q., Yuan C., Yi Z., Zhao M., Wang Z. (2022). Impact of adverse childhood experiences on the symptom severity of different mental disorders: A cross-diagnostic study. General Psychiatry.

[B50-behavsci-15-01044] Haapasalo J., Hämäläinen T. (1996). Childhood family problems and current psychiatric problems among young violent and property offenders. Journal of the American Academy of Child & Adolescent Psychiatry.

[B51-behavsci-15-01044] Hamed A. M., Kauer A. J., Stevens H. E. (2015). Why the diagnosis of attention deficit hyperactivity disorder matters. Frontiers in Psychiatry.

[B52-behavsci-15-01044] Hoza B. (2007). Peer functioning in children with ADHD. Ambulatory Pediatrics.

[B53-behavsci-15-01044] Issa B. A., Yussuf A. D., Ajiboye P. O., Buhari O. I. N. (2009). Prevalence of psychiatric morbidity among inmates of a borstal institution in Nigeria. International Journal of Prisoner Health.

[B49-behavsci-15-01044] Jones W. T., Peters S., Byrne R. E., Shiers D., Law H., Parker S. (2021). “It felt very special, it felt customised to me”—A qualitative investigation of the experiences of participating in a clinical trial of CBT for young people at risk of bipolar disorder. Psychology and Psychotherapy.

[B54-behavsci-15-01044] Kaplan S. G., Cornell D. G. (2004). Psychopathy and ADHD in adolescent male offenders *. Youth Violence and Juvenile Justice.

[B55-behavsci-15-01044] Kganyago Mphaphuli L., Silva T. (2023). The impact of dysfunctional families on the mental health of children. Education and human development.

[B56-behavsci-15-01044] Khanna D., Shaw J., Dolan M., Lennox C. (2014). Does diagnosis affect the predictive accuracy of risk assessment tools for juvenile offenders: Conduct disorder and attention deficit hyperactivity disorder *. Journal of Adolescence.

[B57-behavsci-15-01044] Knouse L. E., Zvorsky I., Safren S. A. (2013). Depression in adults with attention-deficit/hyperactivity disorder (ADHD): The mediating role of cognitive-behavioral factors. Cognitive Therapy and Research.

[B58-behavsci-15-01044] Laajasalo T., Aaltonen M., Pitkänen J., Ellonen N., Martikainen P. (2025). Adverse childhood experiences (ACEs) and juvenile violent delinquency in multiple successive birth cohorts. Nordic Journal of Criminology.

[B59-behavsci-15-01044] Lindblad F., Isaksson J., Heiskala V., Koposov R., Ruchkin V. (2020). Comorbidity and behavior characteristics of russian male juvenile delinquents with ADHD and conduct disorder *. Journal of Attention Disorders.

[B60-behavsci-15-01044] Lockwood C., Munn Z., Porritt K. (2015). Qualitative research synthesis: Methodological guidance for systematic reviewers utilizing meta-aggregation. International Journal of Evidence-Based Healthcare.

[B61-behavsci-15-01044] Loeber R., Green S. M., Keenan K., Lahey B. B. (1995). Which boys will fare worse? Early predictors of the onset of conduct disorder in a six-year longitudinal study. Journal of the American Academy of Child and Adolescent Psychiatry.

[B62-behavsci-15-01044] Mannuzza S., Klein R. G., Moulton J. L. (2008). Lifetime criminality among boys with attention deficit hyperactivity disorder: A prospective follow-up study into adulthood using official arrest records. Psychiatry Research.

[B63-behavsci-15-01044] Margari L., Margari F., Craig F., La Tegola D., Matera E., Lamanna A. L., Carabellese F., Lecce A. (2015). Psychopathology, symptoms of attention-deficit/hyperactivity disorder, and risk factors in juvenile offenders *. Neuropsychiatric Disease and Treatment.

[B64-behavsci-15-01044] Mattiassich-Szokoli E., Sófi G. (2022). A gyermek- és serdülőkori figyelemhiányos hiperaktivitás (ADHD) okozta nemzetgazdasági problémák és azok lehetséges kezelése. IME—Az Egészségügyi Vezetők Szaklapja.

[B65-behavsci-15-01044] Maurer J. M., Paul S., Anderson N. E., Nyalakanti P. K., Kiehl K. A. (2020). Youth with elevated psychopathic traits exhibit structural integrity deficits in the uncinate fasciculus. NeuroImage: Clinical.

[B66-behavsci-15-01044] Mayer J. S., Brandt G. A., Medda J., Basten U., Grimm O., Reif A., Freitag C. M. (2022). Depressive symptoms in youth with ADHD: The role of impairments in cognitive emotion regulation. European Archives of Psychiatry and Clinical Neuroscience.

[B67-behavsci-15-01044] Mazerolle P., Burton V. S., Cullen F. T., Evans T. D., Payne G. L. (2000). Strain, anger, and delinquent adaptations specifying general strain. Journal of Criminal Justice.

[B68-behavsci-15-01044] Moffitt T. E. (1990). Juvenile delinquency and attention deficit disorder: Boys’ developmental trajectories from age 3 to age 15. Child Development.

[B69-behavsci-15-01044] Moffitt T. E. (2003). Life-course-persistent and adolescence-limited antisocial behavior: A 10-year research review and a research agenda. Causes of conduct disorder and juvenile delinquency.

[B70-behavsci-15-01044] Moffitt T. E., Caspi A. (2001). Childhood predictors differentiate life-course persistent and adolescence-limited antisocialpathways among males and females. Development and Psychopathology.

[B71-behavsci-15-01044] Moher D., Shamseer L., Clarke M., Ghersi D., Liberati A., Petticrew M., Shekelle P., Stewart L. A., PRISMA-P Group (2015). Preferred reporting items for systematic review and meta-analysis protocols (PRISMA-P) 2015 statement. Systematic Reviews.

[B72-behavsci-15-01044] Molina B. S. G., Pelham W. E. (2014). Attention-deficit/hyperactivity disorder and risk of substance use disorder: Developmental considerations, potential pathways, and opportunities for research. Annual Review of Clinical Psychology.

[B73-behavsci-15-01044] Moola S., Munn Z., Sears K., Sfetcu R., Currie M., Lisy K., Tufanaru C., Qureshi R., Mattis P., Mu P. (2015). Conducting systematic reviews of association (etiology): The joanna briggs institute’s approach. International Journal of Evidence-Based Healthcare.

[B74-behavsci-15-01044] Moore K. E., Stuewig J. B., Tangney J. P. (2016). The effect of stigma on criminal offenders’ functioning: A longitudinal mediational model. Deviant Behavior.

[B75-behavsci-15-01044] Mordre M., Groholt B., Kjelsberg E., Sandstad B., Myhre A. M. (2011). The impact of ADHD and conduct disorder in childhood on adult delinquency: A 30 years follow-up study using official crime records. BMC Psychiatry.

[B76-behavsci-15-01044] Munene A., James N. (2017). The prevalence of conduct disorder among juvenile delinquents in selected rehabilitation schools in Kenya.

[B77-behavsci-15-01044] Munn Z., Barker T. H., Moola S., Tufanaru C., Stern C., McArthur A., Stephenson M., Aromataris E. (2020). Methodological quality of case series studies: An introduction to the JBI critical appraisal tool. JBI Evidence Synthesis.

[B78-behavsci-15-01044] Olashore A. A., Akanni O. O., Olashore O. O. (2017). Associate factors of delinquency among incarcerated male juveniles in a borstal institution in Nigeria. International Journal of Forensic Mental Health.

[B79-behavsci-15-01044] Philipp-Wiegmann F., Rösler M., Clasen O., Zinnow T., Retz-Junginger P., Retz W. (2018). ADHD modulates the course of delinquency: A 15-year follow-up study of young incarcerated man *. European Archives of Psychiatry and Clinical Neuroscience.

[B80-behavsci-15-01044] Poon K., Suk-Han Ho C. (2015). Contrasting psychosocial outcomes in Chinese delinquent adolescents with attention deficit and hyperactivity disorder symptoms and/or reading disability *. The Journal of Forensic Psychiatry & Psychology.

[B81-behavsci-15-01044] Pratt T. C., Cullen F. T., Blevins K. R., Daigle L., Unnever J. D. (2002). The relationship of attention deficit hyperactivity disorder to crime and delinquency: A meta-analysis. International Journal of Police Science & Management.

[B82-behavsci-15-01044] Retz W., Retz-Junginger P., Hengesch G., Schneider M., Thome J., Pajonk F.-G., Salahi-Disfan A., Rees O., Wender P. H., Rösler M. (2004). Psychometric and psychopathological characterization of young male prison inmates with and without attention deficit/hyperactivity disorder *. European Archives of Psychiatry and Clinical Neurosciences.

[B83-behavsci-15-01044] Retz W., Rösler M. (2010). Association of ADHD with reactive and proactive violent behavior in a forensic population. ADHD Attention Deficit and Hyperactivity Disorders.

[B84-behavsci-15-01044] Richards K. (2011). What makes juvenile offenders different from adult offenders?.

[B85-behavsci-15-01044] Royal College of Psychiatrists (2023). “Blame it on the brain”: Exploring ADHD as a criminogenic factor.

[B86-behavsci-15-01044] Rösler M., Retz W., Retz-Junginger P., Hengesch G., Schneider M., Supprian T., Schwitzgebel P., Pinhard K., Dovi–Akue N., Wender P., Thome J. (2004). Prevalence of attention deficit–/hyperactivity disorder (ADHD) and comorbid disorders in young male prison inmates *. European Archives of Psychiatry and Clinical Neuroscience.

[B87-behavsci-15-01044] Rösler M., Retz W., Yaqoobi K., Burg E., Retz-Junginger P. (2009). Attention deficit/hyperactivity disorder in female offenders: Prevalence, psychiatric comorbidity and psychosocial implications. European Archives of Psychiatry and Clinical Neuroscience.

[B88-behavsci-15-01044] Rutten A. X., Kempes M., Bongers I. L., Vermeiren R. R. J. M., Van Nieuwenhuizen C. (2022). Offence type and neurodiversity: A comparison of 12–17-year-old boys charged with a criminal offence by diagnosis of autism spectrum disorder, attention deficit hyperactivity disorder or both *. Criminal Behaviour and Mental Health.

[B89-behavsci-15-01044] Sampson R. J., Laub J. H. (1993). Crime in the making: Pathways and turning points through life. Crime & Delinquency.

[B90-behavsci-15-01044] Sarver D. E., McCart M. R., Sheidow A. J., Letourneau E. J. (2014). ADHD and risky sexual behavior in adolescents: Conduct problems and substance use as mediators of risk *. Journal of Child Psychology and Psychiatry.

[B91-behavsci-15-01044] Savolainen J., Hurtig T. M., Ebeling H. E., Moilanen I. K., Hughes L. A., Taanila A. M. (2010). Attention deficit hyperactivity disorder (ADHD) and criminal behaviour: The role of adolescent marginalization. European Journal of Criminology.

[B92-behavsci-15-01044] Saylor K. E., Amann B. H. (2016). Impulsive aggression as a comorbidity of attention-deficit/hyperactivity disorder in children and adolescents. Journal of Child and Adolescent Psychopharmacology.

[B93-behavsci-15-01044] Setyanisa A. R., Setiawati Y., Irwanto I., Fithriyah I., Prabowo S. A. (2022). Relationship between parenting style and risk of attention deficit hyperactivity disorder in elementary school children. The Malaysian Journal of Medical Sciences: MJMS.

[B94-behavsci-15-01044] Sibley M. H., Pelham W. E., Molina B. S. G., Gnagy E. M., Waschbusch D. A., Biswas A., MacLean M. G., Babinski D. E., Karch K. M. (2011). The delinquency outcomes of boys with ADHD with and without comorbidity *. Journal of Abnormal Child Psychology.

[B95-behavsci-15-01044] Silva D., Colvin L., Glauert R., Bower C. (2014). Contact with the juvenile justice system in children treated with stimulant medication for attention deficit hyperactivity disorder: A population study *. The Lancet Psychiatry.

[B96-behavsci-15-01044] Smallenburg L. C. S., Spaan P., Grootendorst-van Mil N. H., Bouter D. C., Hoogendijk W. J. G., Kempes M., Roza S. J. (2024). Sex differences in associations between adolescent psychopathology and delinquency. JAACAP Open.

[B97-behavsci-15-01044] Ståhlberg O., Boman S., Robertsson C., Kerekes N., Anckarsäter H., Nilsson T. (2017). A 3-year follow-up study of Swedish youths committed to juvenile institutions: Frequent occurrence of criminality and health care use regardless of drug abuse *. International Journal of Law and Psychiatry.

[B98-behavsci-15-01044] Thapar A., Van Den Bree M., Fowler T., Langley K., Whittinger N. (2006). Predictors of antisocial behaviour in children with attention deficit hyperactivity disorder. European Child & Adolescent Psychiatry.

[B99-behavsci-15-01044] Van Ameringen M., Mancini C., Simpson W., Patterson B. (2011). Adult attention deficit hyperactivity disorder in an anxiety disorders population: ADHD in anxiety disorders. CNS Neuroscience & Therapeutics.

[B100-behavsci-15-01044] Wojciechowski T. W. (2021). The role of ADHD in predicting the development of violent behavior among juvenile offenders: Participation versus frequency *. Journal of Interpersonal Violence.

[B101-behavsci-15-01044] You Y., Oginni O. A., Rijsdijk F. V., Lim K. X., Zavos H. M. S., McAdams T. A. (2024). Exploring associations between ADHD symptoms and emotional problems from childhood to adulthood: Shared aetiology or possible causal relationship?. Psychological Medicine.

[B102-behavsci-15-01044] Young S., Adamo N., Ásgeirsdóttir B. B., Branney P., Beckett M., Colley W., Cubbin S., Deeley Q., Farrag E., Gudjonsson G., Hill P., Hollingdale J., Kilic O., Lloyd T., Mason P., Paliokosta E., Perecherla S., Sedgwick J., Skirrow C., Woodhouse E. (2020). Females with ADHD: An expert consensus statement taking a lifespan approach providing guidance for the identification and treatment of attention-deficit/ hyperactivity disorder in girls and women. BMC Psychiatry.

[B103-behavsci-15-01044] Young S., Cocallis K. (2021). ADHD and offending. Journal of Neural Transmission.

[B104-behavsci-15-01044] Zulauf C. A., Sprich S. E., Safren S. A., Wilens T. E. (2014). The complicated relationship between attention deficit/hyperactivity disorder and substance use disorders. Current Psychiatry Reports.

